# The Drug-Induced Interface That Drives HIV-1 Integrase Hypermultimerization and Loss of Function

**DOI:** 10.1128/mbio.03560-22

**Published:** 2023-02-06

**Authors:** Matthew R. Singer, Tung Dinh, Lev Levintov, Arun S. Annamalai, Juan S. Rey, Lorenzo Briganti, Nicola J. Cook, Valerie E. Pye, Ian A. Taylor, Kyungjin Kim, Alan N. Engelman, Baek Kim, Juan R. Perilla, Mamuka Kvaratskhelia, Peter Cherepanov

**Affiliations:** a Chromatin Structure & Mobile DNA Laboratory, The Francis Crick Institute, London, United Kingdom; b Division of Infectious Diseases, School of Medicine, University of Colorado, Aurora, Colorado, USA; c Department of Chemistry and Biochemistry, University of Delaware, Newark, Delaware, USA; d Macromolecular Structure Laboratory, The Francis Crick Institute, London, United Kingdom; e ST Pharm Co. Ltd., Seoul, South Korea; f Department of Cancer Immunology and Virology, Dana-Farber Cancer Institute, Boston, Massachusetts, USA; g Department of Medicine, Harvard Medical School, Boston, Massachusetts, USA; h Center for Drug Discovery, Children’s Healthcare of Atlanta, Atlanta, Georgia, USA; i Department of Pediatrics, School of Medicine, Emory University, Atlanta, Georgia, USA; j Department of Infectious Disease, St-Mary's Campus, Imperial College London, London, United Kingdom; University of Pennsylvania

**Keywords:** antiretroviral drugs, HIV-1, integrase, allosteric inhibitor, ALLINI, LEDGIN, Pirmitegravir, STP0404, BI-D

## Abstract

Allosteric HIV-1 integrase (IN) inhibitors (ALLINIs) are an emerging class of small molecules that disrupt viral maturation by inducing the aberrant multimerization of IN. Here, we present cocrystal structures of HIV-1 IN with two potent ALLINIs, namely, BI-D and the drug candidate Pirmitegravir. The structures reveal atomistic details of the ALLINI-induced interface between the HIV-1 IN catalytic core and carboxyl-terminal domains (CCD and CTD). Projecting from their principal binding pocket on the IN CCD dimer, the compounds act as molecular glue by engaging a triad of invariant HIV-1 IN CTD residues, namely, Tyr226, Trp235, and Lys266, to nucleate the CTD-CCD interaction. The drug-induced interface involves the CTD SH3-like fold and extends to the beginning of the IN carboxyl-terminal tail region. We show that mutations of HIV-1 IN CTD residues that participate in the interface with the CCD greatly reduce the IN-aggregation properties of Pirmitegravir. Our results explain the mechanism of the ALLINI-induced condensation of HIV-1 IN and provide a reliable template for the rational development of this series of antiretrovirals through the optimization of their key contacts with the viral target.

## INTRODUCTION

Highly active antiretroviral therapy has transformed HIV-1 infection from a terminal illness to a manageable condition with a near normal life expectancy. The combinatorial treatment approach relies on multiple drugs with independent modes of action, typically targeting two or three viral enzymes. A lack of drug supply and/or insufficient adherence leads to the emergence of drug resistance and virologic failure. Infection with drug-resistant HIV-1 strains may then greatly limit treatment options. Therefore, to stay ahead of viral resistance, it is important to continue developing established as well as novel classes of antiretrovirals.

Integrase (IN) is the retroviral enzyme that is responsible for the insertion of the DNA copy of the viral RNA genome into a host cell chromosome (reviewed in [1]). As such, it is a target for strand transfer inhibitors, which are a class of small molecules that bind in the IN active site and arrest HIV-1 replication (2, 3). Five strand transfer inhibitors, including the long-acting second-generation compound cabotegravir, have been approved for the treatment of HIV-1 infection. Due to their shared mode of action, strand transfer inhibitors display overlapping resistance profiles (4–7). Allosteric HIV-1 IN inhibitors (ALLINIs) are an emerging class of antiretrovirals with an orthogonal mode of action. These small molecules were discovered and optimized by independent groups that used different approaches. Consequently, they are also known as LEDGF-IN site inhibitors (LEDGINs) (8), IN-LEDGF allosteric inhibitors (INLAIs) (9, 10), multimerization IN inhibitors (MINIs) (11), or noncatalytic IN inhibitors (NCINIs) (12, 13).

ALLINIs bind with high affinity in a small pocket at the HIV-1 IN catalytic core domain (CCD) dimer interface (8, 9, 11, 14–18). This pocket is also involved in the interaction with the host protein LEDGF/p75 that directs lentiviral DNA integration to bodies of active transcription units (19–22). Consequently, ALLINIs compete with LEDGF/p75 for binding to HIV-1 IN (8, 10, 23). Although gene-tropic HIV-1 integration can accordingly be suppressed in the presence of ALLINIs (23–25), these compounds more potently disrupt the viral life cycle at the stage of particle morphogenesis (10, 15, 26, 27). When HIV-1 virions are produced in the presence of ALLINIs, the genomic RNA is mislocalized outside the viral core (10–12, 15, 26–28), and, upon their entry into target cells, these virions are unable to complete reverse transcription (11, 12, 15, 26, 28, 29). Strikingly similar phenotypes are displayed by the class II HIV-1 IN mutant viruses (30, 31). Unlike class I HIV-1 IN mutants, which are typified by amino acid substitutions within the IN active site and are specifically blocked for integration, class II mutants are characterized by pleiotropic defects and are not confined to a specific IN region or domain. Recent evidence has indicated that HIV-1 IN in the presence of ALLINIs, as well as class II IN mutant proteins, are compromised for RNA binding, which is a noncatalytic function of HIV-1 IN that is essential for viral morphogenesis (31–33). Plausibly, the disruption of IN-RNA interactions may be responsible for the mislocalization of the viral genomic material within affected virions.

**FIG 1:**
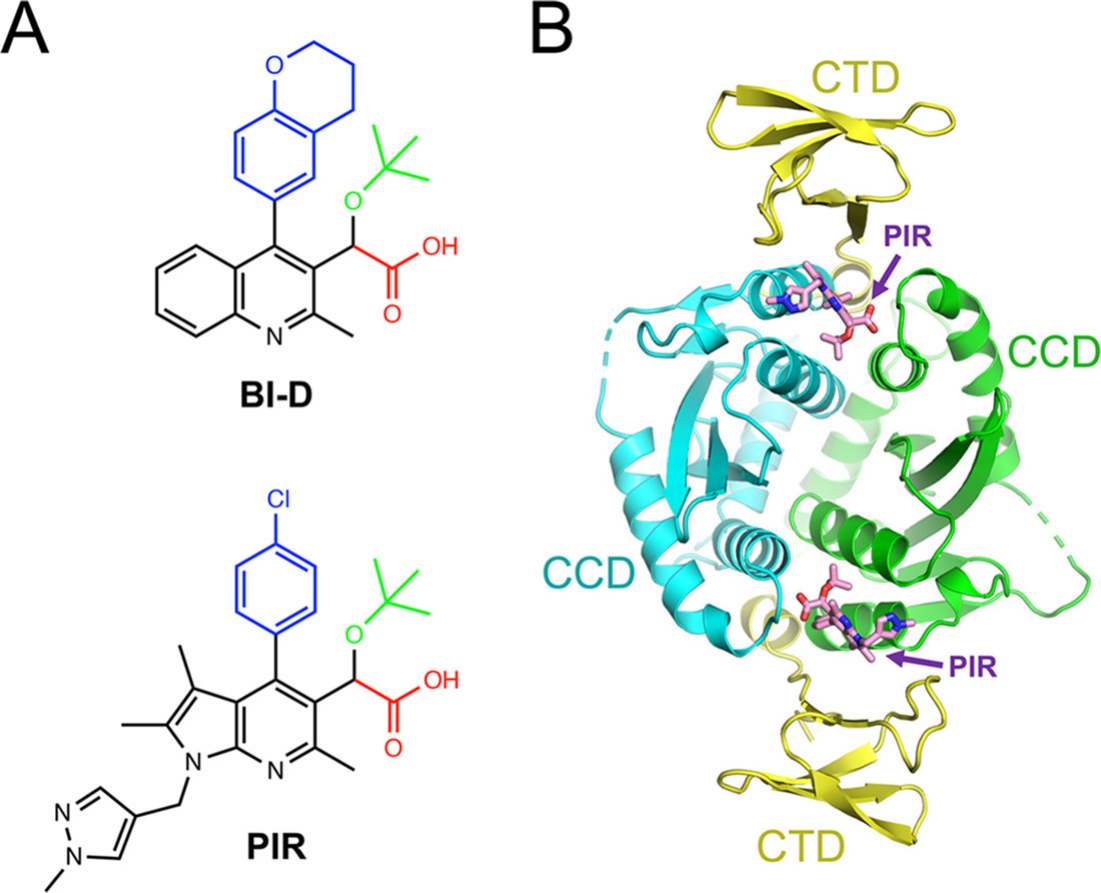
Cocrystallization of HIV-1 IN with ALLINIs. (A) Chemical structures of BI-D (upper) and PIR (lower). The functional groups in the ALLINI pharmacophore are color-coded: carboxyl groups (red); *tert*-butoxyl (green); bulky hydrophobic side chains, chromanyl of BI-D, and chlorophenyl of PIR (blue). Central aromatic scaffolds (quinoline in BI-D and pyrrolopyridine in PIR) are black. (B) Structure of the HIV-1 IN-PIR complex. The protein backbone is shown in a cartoon representation, with CTDs in yellow and CCD dimer chains in cyan and green. The ALLINI molecules are shown as sticks, with carbon, nitrogen, and oxygen atoms in pink, blue, and red, respectively.

ALLINIs trigger aberrant HIV-1 IN multimerization within virions as well as the rapid aggregation of the recombinant protein *in vitro* (10, 15, 26). This property relies on an unnatural, inhibitor-induced intermolecular interaction that involves the dimeric CCD of one IN oligomer as well as the carboxyl-terminal domain (CTD) of a separate IN multimer (16, 34). Under physiological conditions, lentiviral INs have the propensity to form tetramers and larger order functional oligomers in the presence of viral DNA and are stabilized by the homomeric dimerization of the CCD and the CTD (35–40). However, by inducing the additional CTD-CCD interface, the compounds promote the uncontrolled propagation of linear and branched IN polymers (41). The principal ALLINI binding pocket at the HIV-1 IN CCD dimer interface has been well-characterized via X-ray crystallography, and the crystal structures were instrumental in the development of these compounds (8, 10, 11, 15). However, the ALLINI-induced CTD-CCD interface has only been visualized at 4.4 Å resolution (16), which was insufficient to explain the role of the small molecules in its formation. The shared ALLINI functionality, which is composed of carboxyl, *tert*-butoxyl, and bulky hydrophobic groups (Fig. 1A), has been visualized at a comparatively high resolution in HIV-1 IN CCD dimer cocrystal structures. However, the apparent lack of similarity outside these CCD-binding moieties may appear inconsistent with their mechanism of action, which requires the least conserved portion of the chemical structure to serve a shared role. Here, we describe the high-resolution cocrystal structures of a two-domain HIV-1 IN construct with two highly potent ALLINIs, namely, BI-D (10, 15, 42, 43) and the current drug candidate Pirmitegravir (PIR; originally designated STP0404) (17). The structures reveal atomistic details of the ALLINI-induced CTD-CCD interface and provide reliable means for the further optimization of this promising class of antiretrovirals.

## RESULTS

### HIV-1 IN-ALLINI cocrystal structures.

The rapid aggregation of HIV-1 IN in the presence of ALLINIs greatly impedes the cocrystallization of the viral protein with these small molecules. To overcome this obstacle, we sought to design novel HIV-1 IN constructs with a reduced propensity for aggregation, while preserving the key components for ALLINI interactions. HIV-1 IN is comprised of three flexibly linked globular domains, the N-terminal domain (NTD), CCD, and CTD, each of which is involved in the functional oligomerization of the full-length protein (31). In particular, CTD dimerization (36, 39) is crucial for the formation of higher-order lentiviral IN oligomers (40, 44). Moreover, the CTD dimer interface contributes to the propagation of ALLINI-induced aberrant polymers of HIV-1 IN but not to the interaction with ALLINIs (41). Therefore, we evaluated the oligomerization properties of HIV-1 CTD variants harboring single amino acid substitutions within the dimerization interface. Multiangle laser light scattering coupled with size exclusion chromatography (SEC-MALLS) revealed that whereas the isolated HIV-1 IN CTD existed in a monomer-dimer equilibrium, the mutants L242E and W243E behaved as monomeric species (Fig. S1A).

10.1128/mbio.03560-22.1 FIG S1 Development, validation, and X-ray crystallography of the HIV-1 IN construct in complex with ALLINIs. (A) Disruption of HIV-1 IN CTD dimerization by a single amino acid substitution. Wild type (left), L242E (middle), or W243E (right) HIV-1 IN CTD constructs were analyzed using SEC-MALLS. Solid lines report the refractive index for each elution, and the scatter points are weight-averaged molar masses that were determined at 1 s intervals throughout the elution of the observed peaks. The colors correspond to loading protein concentrations of 8 (red), 4 (magenta), 2 (green), 1 (blue), or 0.5 (puce) mg/mL. Grey dashes indicate the molecular masses corresponding to the CTD monomer (6.0 kDa) and dimer (12 kDa) species. (B) The CTD enhances the affinity of HIV-1 IN for PIR. SPR sensorgrams of the association and dissociation of PIR from HIV-1 IN CCD (F185H) (left) and CTD-CCD (F185K/W243E) (right). Compound was injected at concentrations of 0.015 to 1 μM, as indicated. The lower panel shows the association and dissociation rate constants (*k_on_* and *k_off_*), the equilibrium dissociation constants (*K_D_*) that were determined from the SPR data, and the fold change between the parameters between the two IN constructs. (C) Electron density maps of the BI-D (left) and PIR (right) cocrystal structures. Shown are wall-eyed stereo views of the ALLINI-induced CTD-CCD interface with final weighted *2Fo-Fc* maps that are contoured at a 1.0 rms deviation as a blue mesh. Protein residues and ligands are depicted as sticks, and water molecules are depicted as small red spheres. The carbon atoms of the amino acid residues are colored by chain (CTD, yellow; subunits 1 and 2 of the CCD dimer, cyan and green, respectively). The carbon atoms of the ALLINIs are colored pink. The carbon atoms of PEG and ethylene glycol are colored grey. The remaining atoms are colored according to conventions: oxygen, red; nitrogen, blue. Download FIG S1, PDF file, 3.1 MB. Copyright © 2023 Singer et al. 2023 Singer et al. https://creativecommons.org/licenses/by/4.0/ This content is distributed under the terms of the Creative Commons Attribution 4.0 International license.

Having tested a range of recombinant HIV-1 IN constructs, we focused on a reverse-oriented CTD-CCD variant that spanned the CTD with the dimer-disrupting substitution W243E, which was in turn linked via a flexible peptide to the CCD harboring the solubilization mutation F185K (35, 44). The linker was comprised of the HIV-1 IN 18-residue C-terminal tail region, which is unstructured (31), fused to 6 residues of the flexible NTD-CCD linker. The resulting construct lacked the NTD, which is involved in lentiviral IN tetramerization (38, 45) but does not contribute to ALLINI binding (41). Our design was inspired by the idea that linking the domains via a flexible sequence could promote intramolecular CTD-ALLINI-CCD contacts and thus limit the aggregation of the IN-drug assemblies.

Surface plasmon resonance (SPR) measurements revealed that the CTD-CCD fusion construct bound PIR, a clinical candidate ALLINI (17), with an approximately 30-fold higher affinity and an approximately 10-fold slower dissociation rate than did the isolated HIV-1 IN CCD (Fig. S1B). Moreover, the affinity of PIR for the CTD-CCD fusion (*K_D_* ≈ 5 nM) closely correlated with the antiviral activity of the ALLINI, which displayed a 50% effective concentration (EC_50_) of approximately 12 nM to inhibit HIV-1 infection (see below) (17). Collectively, these results are concordant with the key involvement of the CTD in the formation of HIV-1 IN-ALLINI complexes (16, 34) and validate the ability of our engineered CTD-CCD protein to meaningfully interact with the small molecules.

Following extensive screening, we were able to cocrystallize the CTD-CCD fusion with two highly potent ALLINIs: BI-D (10, 15, 42, 43) and PIR (17) (Fig. 1A). The BI-D and PIR containing crystals diffracted X-rays to the resolution of 1.8 and 2.1 Å, respectively (Table S1). The asymmetric units contained a canonical CCD dimer with two associated CTDs, each of which sandwiched an ALLINI molecule (Fig. 1B; Fig. S1C and S2A). 22 and 15 residues of the interdomain linker region were disordered in the BI-D and PIR cocrystals, respectively, suggesting that the linker was free to adopt variable conformations throughout the crystal lattices. Therefore, it is unlikely that the artificial linkage affected the well-ordered portions of the protein, including the critical CTD-CCD interface. The four independently observed CTD-CCD interfaces in the crystal structures were highly similar (Fig. S2A). Moreover, the observed relative positioning of the CTDs and CCD dimers in our structures correspond to those in the 4.4 Å resolution cocrystal structure of full-length HIV-1 IN with ALLINI GSK1264 (PDB entry 5HOT) (Fig. S2B and C) (16), further validating our crystallography strategy. Pairwise alignments of the CCD dimers in our crystal structures resulted in rms deviations between CTD Cα atom positions of 2.2 ± 0.7 Å, whereas the same metric for the CTD β1 and β5 strands that are directly involved in the interface was 1.2 ± 0.5 Å (Fig. S2C). Thus, the ALLINI-induced interface allows for minor relative adjustments of the CTD and the CCD dimer.

10.1128/mbio.03560-22.2 FIG S2 Conservation of the ALLINI-induced CTD-CCD interface in the crystal structures. (A) Superposition of the two crystal structures determined in this study. Protein backbones are shown as cartoons, with CTDs in yellow and CCD dimer chains in cyan and green. The ALLINI molecules (indicated with arrowheads) are shown as sticks, with carbon, nitrogen, and oxygen atoms in pink, blue, and red, respectively. (B) Left panel: a linear polymer of HIV-1 IN dimers observed in a cocrystal structure with GSK1264 (PDB entry 5HOT) is stabilized by ALLINI-induced association of the CCD dimers with CTDs provided by neighboring dimers. An example of this substructure is boxed. Right panel: the CCD dimer with its associated CTD chains is shown in the same orientation as the crystal structures in panel A. (C) Left panel: rhe four independent CTD-ALLINI-CCD interfaces that were observed in the crystal structures that are reported here as well as the related substructure from 5HOT were superposed, based on the CCD dimers. The CTDs from cocrystal structures with BI-D, PIR, and GSK1264 are shown as ribbons and are colored orange, yellow, and brown, respectively. Right panel: the superposition of the CTDs from the cocrystal structures with BI-D (orange) and PIR (yellow) with a prior drug-free HIV-1 IN CTD structure (PDB entry 6T6E). Individual β strands of the five-bladed SH3 fold are indicated (β1 to β5). Note that the CTD structure does not undergo substantial conformation changes as a result of ALLINI binding. Download FIG S2, PDF file, 0.6 MB. Copyright © 2023 Singer et al. 2023 Singer et al. https://creativecommons.org/licenses/by/4.0/ This content is distributed under the terms of the Creative Commons Attribution 4.0 International license.

10.1128/mbio.03560-22.10 TABLE S1 X-ray crystallography statistics and free energy perturbation data. Download Table S1, XLSX file, 0.02 MB. Copyright © 2023 Singer et al. 2023 Singer et al. https://creativecommons.org/licenses/by/4.0/ This content is distributed under the terms of the Creative Commons Attribution 4.0 International license.

### The ALLINI-induced CTD-CCD interface.

**FIG 2:**
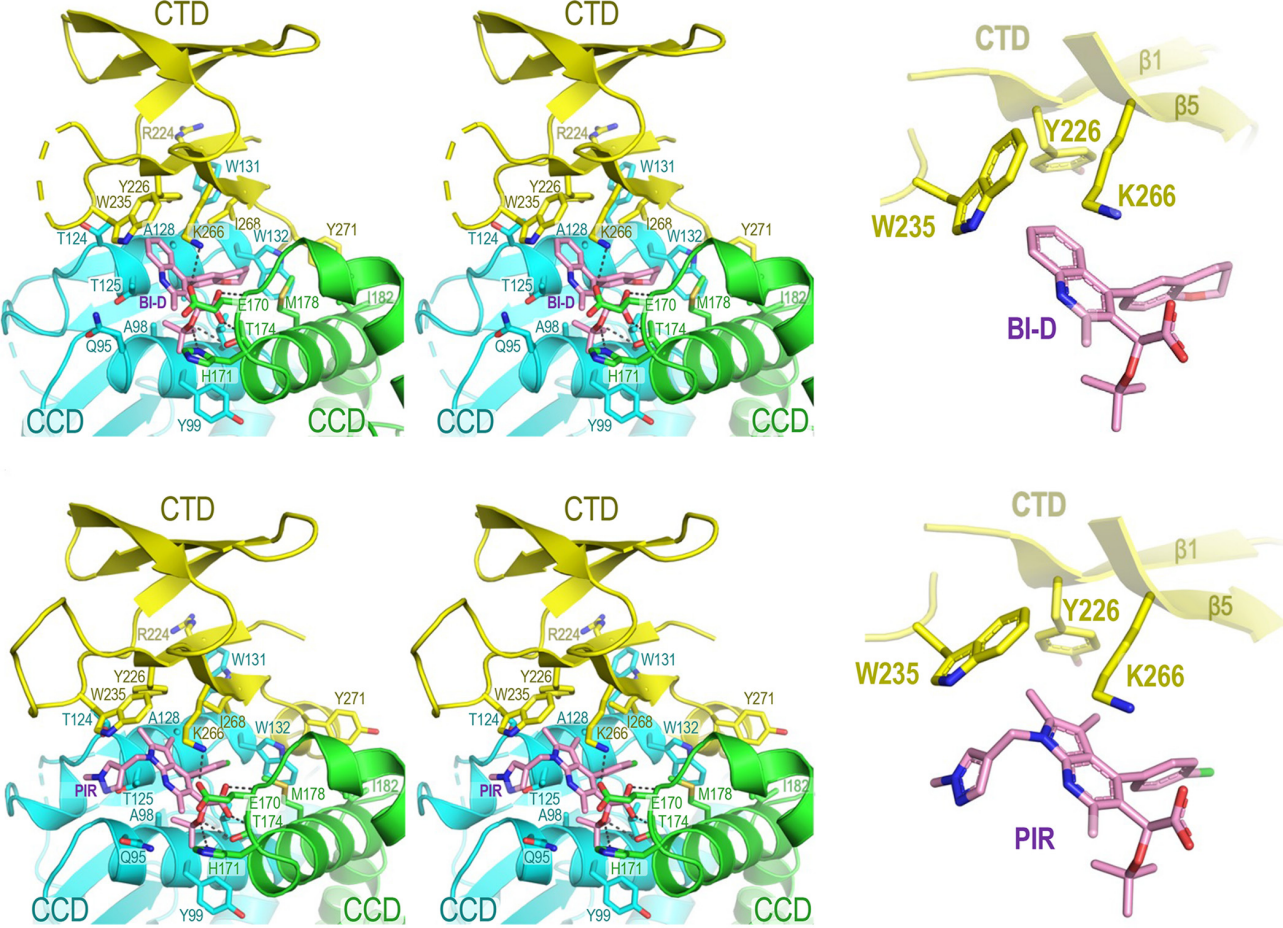
Details of the IN-ALLINI interface. The CTD-CCD interface observed in cocrystals of BI-D (upper) and PIR (lower) IN complexes. The left panels show stereo wall-eyed views of the interfaces, and the right panels show the key CTD residues involved in the stacking interactions with the ALLINIs. Selected amino acid residues, along with the inhibitor molecules, are labeled and shown as sticks. Black dashes on the left panels indicate hydrogen bonds. In the right panels, the aromatic and lysine residue side chains that make delocalized interactions with inhibitor molecules are shown.

**FIG 3:**
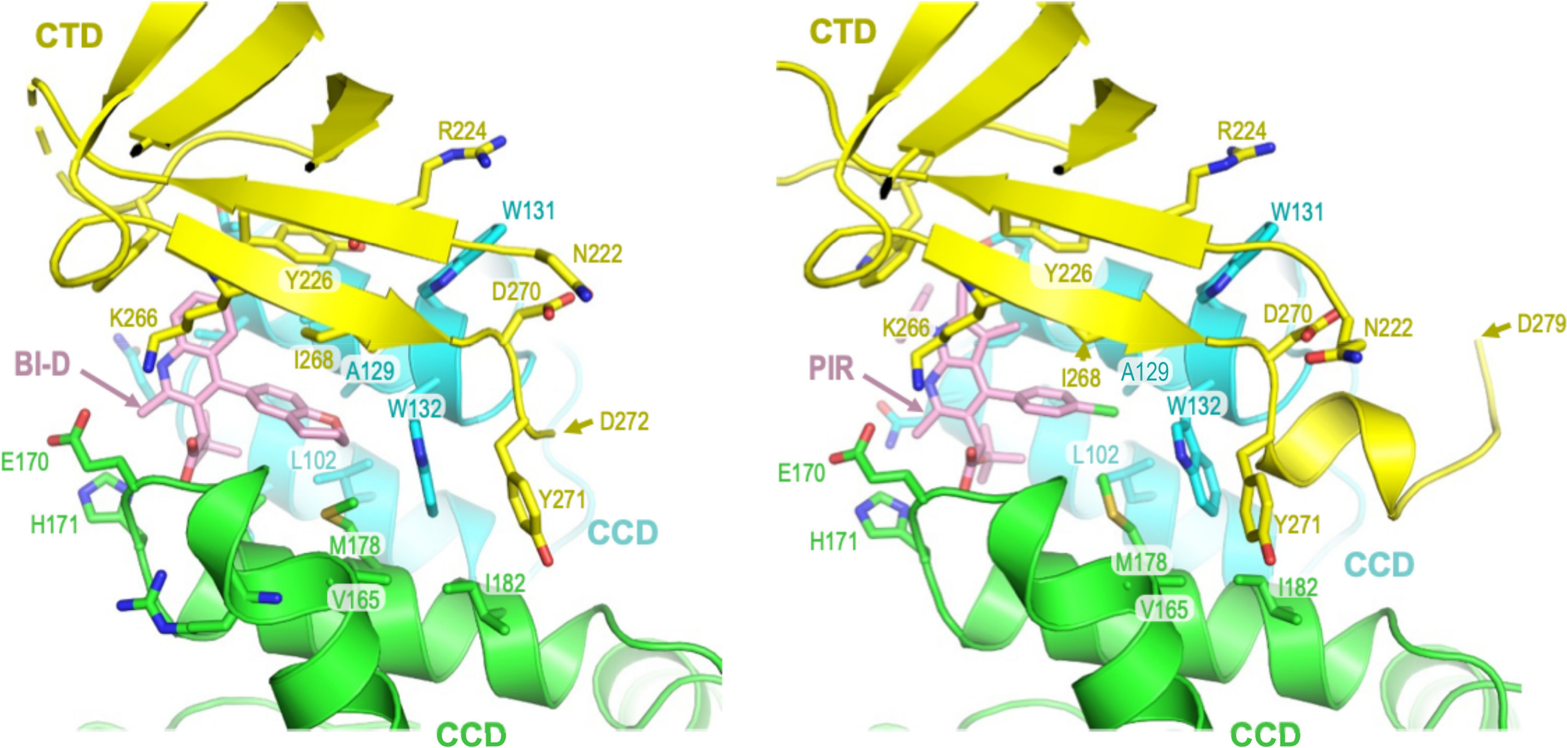
ALLINI-induced CTD-CCD contacts outside the immediate vicinity of the bound inhibitors. The last residues that originate from the HIV-1 IN C-terminal tail region in the refined models are indicated with arrowheads (Asp272 and Asp279 in the BI-D and PIR complexes, respectively). Other labels and colorings are the same as in Figure 2.

ALLINIs share three chemical features that are attached to an aromatic scaffold: a bulky hydrophobic group (chromanyl and chlorophenyl in BI-D and PIR, respectively), a *tert*-butoxyl side chain, and an invariant carboxylate (shown in blue, green, and red, respectively, in Fig. 1A). In agreement with prior observations (8, 10, 11, 15, 17), the compounds insert into the principal ALLINI binding pocket at the CCD dimer interface. Within it, the carboxylate group forms a bidentate hydrogen bond with the main chain amides of Glu170 and His171, whereas the *tert*-butoxyl and the bulky aromatic groups make hydrophobic interactions with side chains of both CCD dimer subunits (Gln95, Tyr99, Leu102, Tr125, Trp132, Thr174, and Met178) (Fig. 2 and 3).

**FIG 4:**
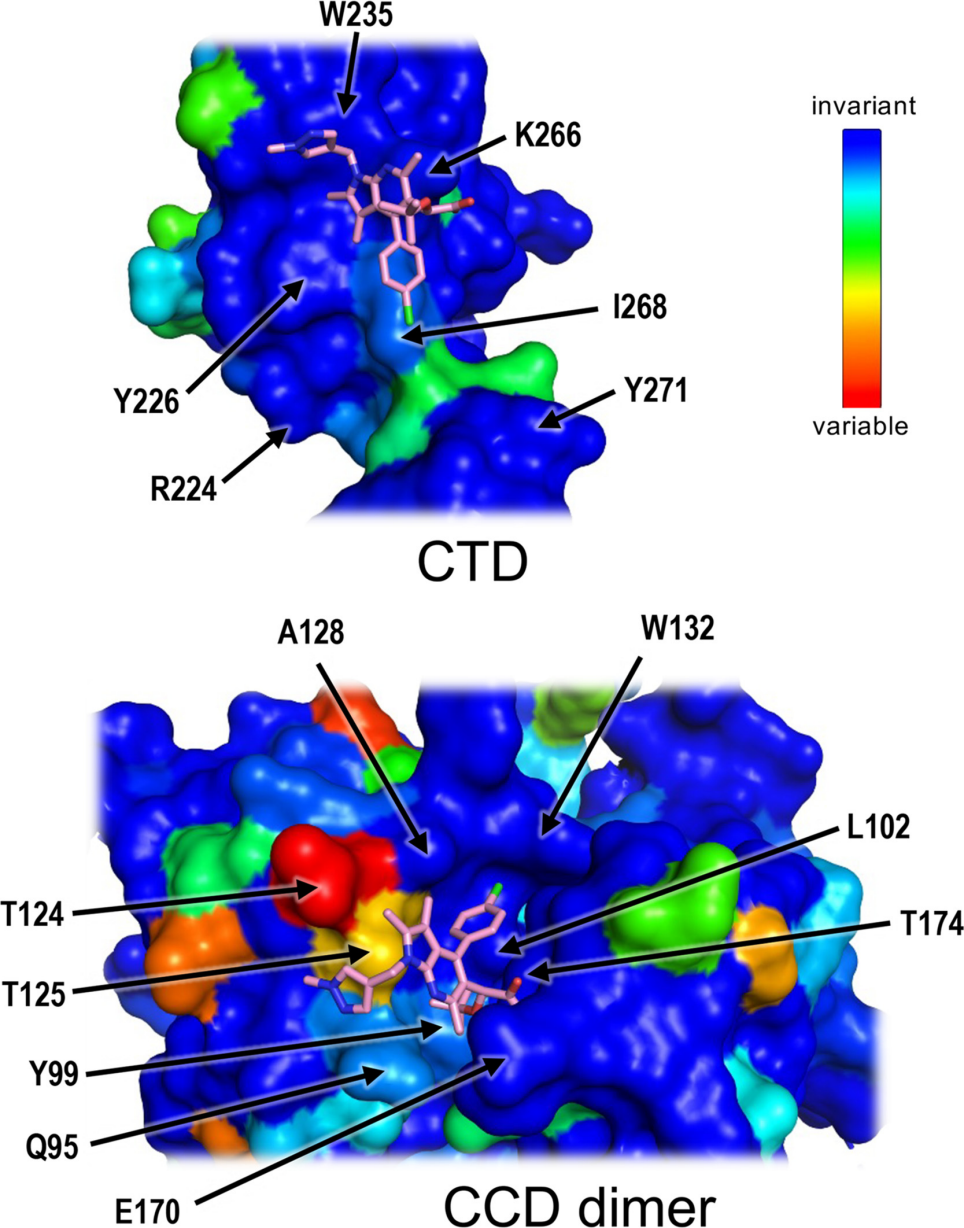
Conservation of HIV-1 IN amino acid residues involved in the ALLINI-induced CTD-CCD interface. Open-book view of the interface with the CTD (upper) and the CCD dimer (lower) shown in surface representations and colored according to amino acid conservation, as shown on the scale: from dark blue (invariant and most conserved) to red (most variable). Selected residues are indicated, and PIR is shown as sticks. The conservation scores were calculated using the ConSurf server (https://consurf.tau.ac.il/) (98) from an alignment of IN amino acid sequences of representative HIV-1 strains, including the subtypes M, N, and O as well as recombinant viruses and simian immunodeficiency viruses from chimpanzees (46).

In addition to these well-characterized ALLINI-CCD interactions, our crystal structures now reveal details of the ALLINI-induced CTD-CCD interface (Fig. 2 and 3). The aromatic scaffolds of the compounds (quinoline in BI-D and pyrrolopyridine in PIR [shown in black in Fig. 1A]) protrude sufficiently from the CCD dimer to make T-shaped π-π stacking interactions with the side chains of the invariant CTD residues Tyr226 and Trp235. Remarkably, this configuration allows the side chain of the invariant CTD residue Lys266 to pack along the corner formed by the aromatic planes of the inhibitor scaffold and Trp235. This orients Lys266 for ionic interactions with the side chain of CCD Glu170 and carboxylate groups of the inhibitors. Additionally, the methylpyrazole side chain in PIR forms a parallel π-π stacking interaction with Trp235. Within the buried CCD dimer interface, the chromanyl of BI-D and the chlorophenyl of PIR engage in hydrophobic interactions with the side chain of CTD Ile268. This residue, although not invariant among circulating HIV-1 strains, is nevertheless highly conserved, with the occasional occurrence of Leu at this position in a minority of HIV-1 isolates (46). Thus, the compounds harness four invariant (Glu170, Tyr226, Lys266, and Trp235) and one highly conserved (Ile268) HIV-1 IN residues to facilitate the CTD-CCD interaction (Fig. 4).

The extent of the ALLINI-induced CTD-CCD interface occludes a total of approximately 1,200 Å^2^ of molecular surface, and it is stabilized by interactions of highly conserved or invariant HIV-1 IN residues outside the direct vicinity of the ALLINIs (Fig. 2 to 4). Overall, the CTD-CCD contacts are devoid of water molecules and are primarily mediated by hydrophobic and π stacking interactions. This includes the side chain of CTD residue Arg224, which forms a cation-π stacking interaction with the invariant CCD residue Trp131 (Fig. 2 and 3). In addition, the invariant CTD residue Tyr271, which is located at the beginning of the C-terminal tail immediately abutting the CTD, makes hydrophobic interactions with CCD residues Val165, Ile182 (both highly conserved), and Trp132 (invariant). The superposition of the CTD chains in our refined models with a crystal structure of the isolated CTD (47) revealed a rms deviation of Cα atom positions of only 0.4 Å (Fig. S2C). Therefore, the formation of the ALLINI-induced interface does not require substantial conformational adaptation of the IN CTD.

### Energetics and dynamics of the ALLINI-induced CTD-CCD interface.

We next applied computational chemistry to quantitatively assess the interactions observed in the cocrystal structures. To this end, we extended the experimental structures by modeling polypeptide loops that were disordered in the crystals (Fig. S3A), placed them in an appropriate box of solvent (Fig. S3B–D), and conducted 1 μs all-atom molecular dynamics (MD) simulations in the absence and presence of BI-D or PIR. Initially, we computed heavy atom rms deviations for the CCD and CTD chains with respect to the first frame of the corresponding simulation (Fig. S4A to C). When the MD was performed in the absence of ALLINIs, the CCD and CTD chains diverged from the initial states by 3.23 ± 0.38 Å and 6.62 ± 1.16 Å, respectively (Fig. S4A). As expected, the ALLINIs strongly stabilized the interface, considerably diminishing the divergence of both domains during the simulations. In particular, the rms deviations of the CTD in the presence of BI-D or PIR reduced to 4.39 ± 0.32 or 3.03 ± 0.30 Å, respectively (Fig. S4B and C). Snapshots of the simulation in the absence of the ALLINIs revealed that each CTD chain underwent partial unfolding and drifted from its initial position (Fig. S4A). In contrast, in the presence of BI-D or PIR, the CTD remained stably engaged with the CCD dimer throughout the MD simulations (Fig. S4B and C). The examination of the rms fluctuations per protein residue further confirmed that the presence of either compound greatly rigidified the CTD-CCD interface (Fig. S4D). To identify the events that were responsible for the comparatively dramatic structural fluctuations observed in the absence of ALLINIs, we performed rms deviation matrix analyses between pairwise structures across the MD trajectory. Figure S4E demonstrates the extents to which the CTDs of the apo form of our construct transitioned through various conformational meta states, including its dislodgement from the CCD dimer.

10.1128/mbio.03560-22.3 FIG S3 MD setup. (A) Modeled loops. BI-D (left) and PIR (right) cocrystal structures completed by modeling the missing polypeptide loops. For clarity, ALLINI molecules are not shown. The protein mainchains are shown as cartoons, with the CCDs in orange, CTDs in red, and modeled loops in blue. (B–D) MD simulation domains. Simulation domains for the systems without ALLINI (B), with BI-D (C), or with PIR (D). Protein chains are shown as cartoons, with CCD in orange and CTD in red. Inhibitors are shown in space-filling representations. The purple, green, and cyan spheres represent sodium, chloride, and Mg^2+^ ions, respectively. The grey boxes are solvent water. (E) An example of the thermodynamic cycle of BI-D binding. The horizontal legs represent the alchemical transformation of an amino acid residue (Lys266) into Ala either in the bound state (complex, upper panel) or in the unbound state (free, bottom panel). The vertical legs represent binding. Download FIG S3, PDF file, 0.3 MB. Copyright © 2023 Singer et al. 2023 Singer et al. https://creativecommons.org/licenses/by/4.0/ This content is distributed under the terms of the Creative Commons Attribution 4.0 International license.

10.1128/mbio.03560-22.4 FIG S4 MD simulation details. (A–C) The rms deviation time traces of all of the protein heavy atoms are shown for the CCDs (green) and CTDs (yellow) throughout the MD simulations that were conducted either in the absence of ALLINIs (A) or in the presence of BI-D (B) or PIR (C). Corresponding snapshots of the protein backbone and bound compounds are shown for each system, with the CCD and the CTD in green and yellow, respectively. BI-D and PIR are presented in space-filling representations. (D) The rms fluctuation per residue trace in the absence of ALLINI (red), in the presence of BI-D (blue), or in the presence of PIR (purple). (E) All-to-all rms deviation matrix analysis. Shown is the rms deviation matrix (81) between the pairwise structures in the MD trajectory of the protein in the absence of ALLINIs. Representative snapshots are shown to the right (I–VI, indicated with arrowheads on the matrix plot) of the protein, highlighting various transitions observed along the MD trajectory. (F) Dynamics of ALLINIs binding to IN. Snapshots are shown from the MD simulations of the CTD-CCD interface in the presence of BI-D (upper) or PIR (lower) at 0, 250, 500, 750, and 1,000 ns of the MD simulations. The ALLINIs and selected side chains are shown in ball-and-stick mode in separate colors and are labelled on the left diagrams that correspond to the initial states (0 ns). Download FIG S4, PDF file, 0.4 MB. Copyright © 2023 Singer et al. 2023 Singer et al. https://creativecommons.org/licenses/by/4.0/ This content is distributed under the terms of the Creative Commons Attribution 4.0 International license.

**FIG 5:**
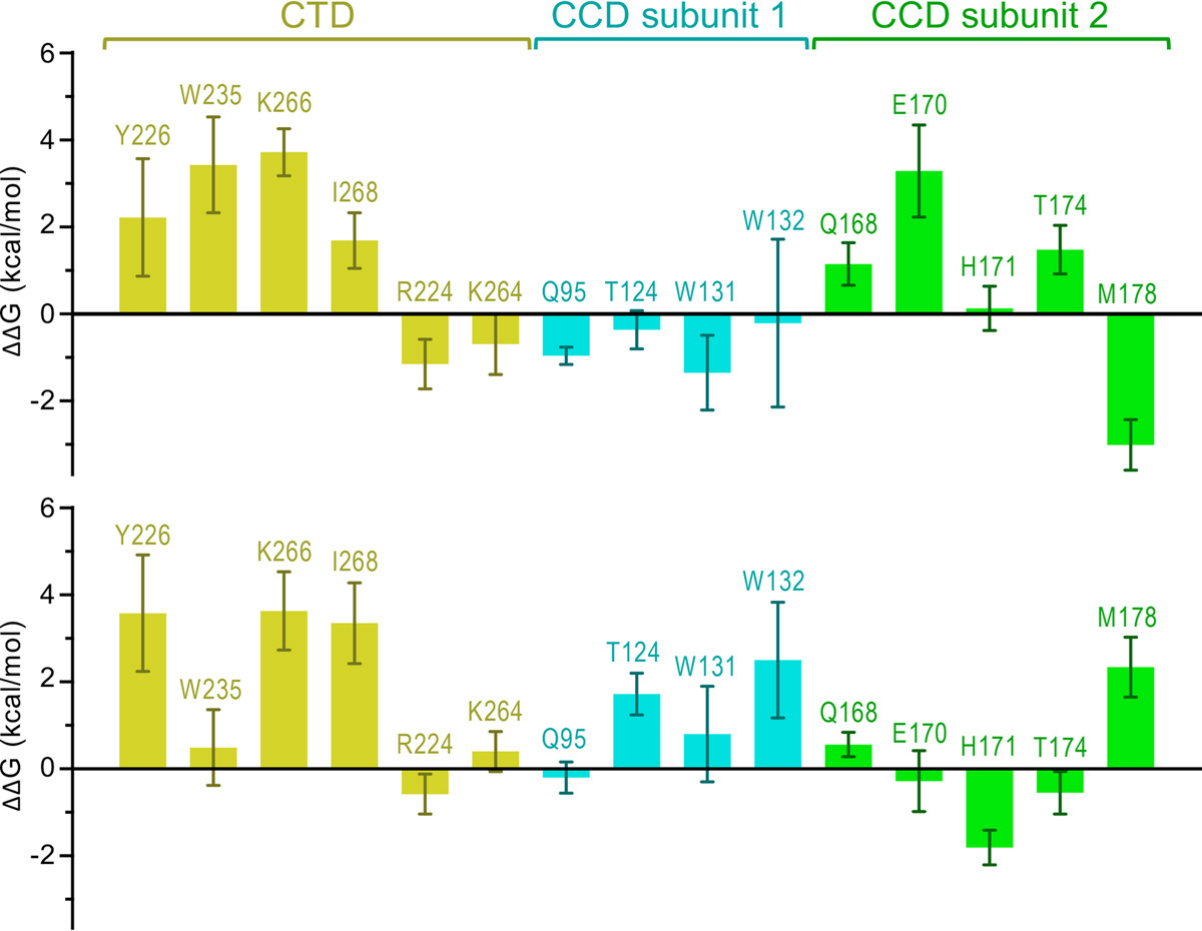
Energetics of ALLINI binding to HIV-1 IN. The calculated relative free energy differences (ΔΔG) of Ala substitutions within the CTD-CCD interface induced by BI-D (upper) or PIR (lower). The error bars represent the standard deviations of the mean values from four independent simulations (Table S1).

Importantly, the results of our MD simulations are in accord with the experimental evidence that ALLINIs stabilize the HIV-1 IN CTD-CCD interface. To quantify the contributions of individual amino acid residues to the ALLINI-induced CTD-CCD interface, we next performed free energy perturbation (FEP) calculations. IN residues that make direct contacts with ALLINIs were alchemically transformed to alanine, and the final free energy differences (ΔΔG) were computed using the thermodynamic cycle (Fig. S3E). Additionally, we computed ΔΔG values for selected IN amino acid residues (Arg224, Trp131, and Lys264) which, while not directly interacting with the ALLINIs, were located within the specified force field cutoff for nonbonded interactions (12 Å). The resulting ΔΔG values are reported in Fig. 5 and Table S1. A positive ΔΔG value indicates that the side chain of the truncated residue has a stabilizing role within the IN-ALLINI interface. Throughout the MD simulations in the presence of BI-D or PIR, Lys266 interacted with the aromatic ALLINI scaffold via cation-π stacking and formed a salt bridge to the inhibitor carboxylate group (Fig. S4F). FEP analyses revealed that the Lys266 side chain had a strong contribution to ALLINI binding, with corresponding ΔΔG values of 3.72 ± 0.54 and 3.63 ± 0.90 kcal/mol for the BI-D and PIR-induced interfaces, respectively. Likewise, the MD simulations confirmed that both BI-D and PIR formed stable T-shaped π-π stacking interactions with the side chain of Tyr226 (Fig. S4F), which accounted for 2.22 ± 1.35 kcal/mol and 3.58 ± 1.34 kcal/mol of binding energy, respectively. Throughout the simulations, the side chain of Ile268 was oriented to form hydrophobic interactions with the chromanyl and chlorophenyl groups of BI-D and PIR, contributing ΔΔG of 1.69 ± 0.64 kcal/mol and 3.35 ± 0.93 kcal/mol, respectively (Fig. 5). Our computations also suggested that the energetic contributions of some of the IN residues may depend on the specific ALLINI. Thus, the side chain of Trp235 engaged in stable T-shaped π-π stacking with BI-D (Fig. S4F), contributing as much as 3.43 ± 1.1 kcal/mol of binding energy. In contrast, the stacking of Trp235 with PIR was less extensive (Fig. S4F), and the substitution of Trp235 with Ala was associated with a ΔΔG of 0.49 ± 0.87 kcal/mol. Likewise, the side chain of the CCD residue Glu170 contributed more binding energy to BI-D (3.29 ± 1.06 kcal/mol) than to PIR (−0.28 ± 0.70 kcal/mol) due to stability of the salt bridge to Lys266 during the MD simulations. Moreover, according to the FEP analysis, we observed that the CCD residues Gln168, Thr174, and Glu170 were favorable for the recognition of BI-D, whereas the CCD residues Met178, Trp132, and Thr124 were favorable for the recognition of PIR (Fig. 5; Table S1). These subtle differences between individual compounds notwithstanding, computational chemistry confirmed the importance of the conserved HIV-1 IN CTD residues Lys266, Trp235, Tyr226, and Ile268 for the interactions with the ALLINIs.

### Site directed mutagenesis of the residues at the CTD-CCD interface.

**FIG 6:**
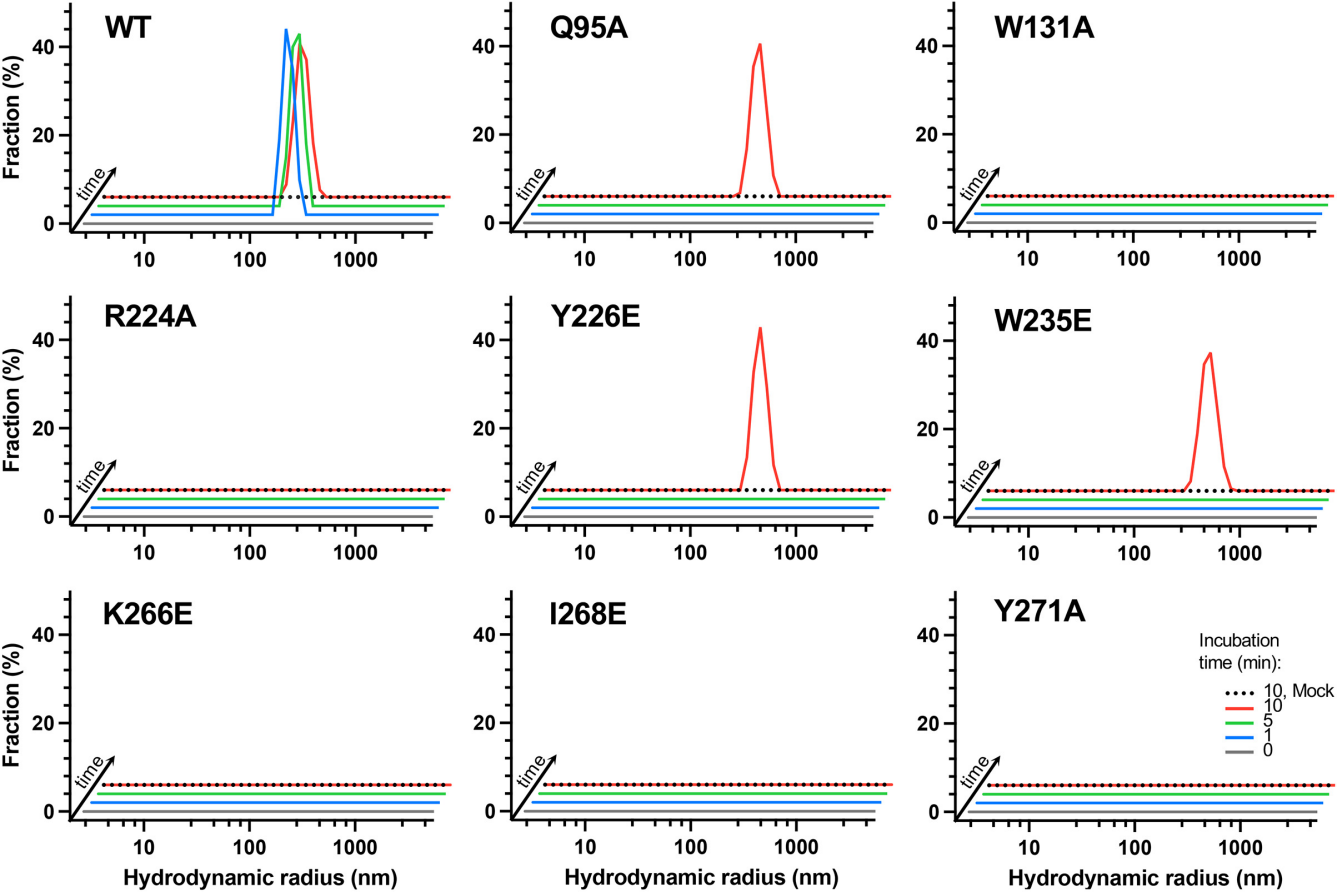
Amino acid substitutions within the ALLINI-induced CTD-CCD interface reduce IN aggregation by PIR. Full-length wild type and mutant IN proteins were incubated in the absence (black dotted lines) or presence of PIR for 0, 1, 5, or 10 min (gray, blue, green, and red lines, respectively). The plots show the distribution of the observed hydrodynamic radii (nm) that were determined via dynamic light scattering.

ALLINIs are known to cause the rapid aggregation of HIV-1 IN (10, 15, 16, 26, 41). Concordantly, the incubation of 4 μM recombinant wild type IN with near stoichiometric amounts (2.5 or 5 μM) of PIR resulted in the near quantitative precipitation of the protein (Fig. S5). Furthermore, dynamic light scattering (DLS) revealed the formation of protein aggregates within minutes of supplementing 12.5 nM HIV-1 IN with 62.5 nM PIR (Fig. 6). To probe the contributions of individual residues positioned at the ALLINI-induced CTD-CCD interface to the inhibitor activity, we prepared full-length HIV-1 IN proteins with the following amino acid substitutions: Q95A, W131A, R224A, Y226A, W235A, K266A, I268A, and Y171A. Because some of these proteins displayed aberrant aggregation (for example, the Y226A and K266A INs precipitated in the absence of an ALLINI [Fig. S5]), we additionally produced Y226E, W235E, K266E, and I268E IN proteins.

10.1128/mbio.03560-22.5 FIG S5 Effects of amino acid substitutions within the CTD-CCD interface on PIR-induced IN aggregation. Full-length wild type and mutant HIV-1 IN proteins (indicated, right) were incubated in the absence or presence of 2.5 or 5 μM PIR. Centrifugally separated precipitated (P) and soluble (S) fractions were analyzed via SDS polyacrylamide electrophoresis, and IN was detected by staining with Coomassie brilliant blue. The migration positions of the protein molecular mass standards are indicated to the left (kDa). Download FIG S5, PDF file, 0.3 MB. Copyright © 2023 Singer et al. 2023 Singer et al. https://creativecommons.org/licenses/by/4.0/ This content is distributed under the terms of the Creative Commons Attribution 4.0 International license.

DLS and analytical size exclusion chromatography analyses confirmed that the Q95A, W131A, R224A, Y226E, W235E, K266E, I268E, and Y271A INs behaved as soluble oligomeric species, although relative amounts of tetramers, dimers, and monomers varied among the variants (Fig. 6; Fig. S5, S6). As revealed by DLS, and in agreement with the cocrystal structures, the mutations suppressed HIV-1 IN aggregation in the presence of PIR (Fig. 6). Although DLS provides a time course for ALLINI-induced IN aggregation, this technique does not delineate what fraction of the initial, soluble protein is converted into higher order aggregates. Therefore, we additionally tested our panel of HIV-1 IN mutants in the ALLINI-induced precipitation assay to monitor both the soluble and the aggregated fractions. Substitutions of IN residues that play key roles in the interface with the inhibitors, namely, Y226E, W235E, K266A, K266E, I268A, and I268E, conferred resistance to precipitation in the presence of PIR (Fig. S5; note that the compound solubilized K266A IN). In contrast, the substitution of Gln95, which plays a relatively minor role at the periphery of the ALLINI-induced CTD-CCD interface (Fig. 2; Table S1), did not reduce ALLINI-mediated precipitation (Fig. S5). The majority of the remaining mutants displayed intermediate phenotypes. Thus, whereas W131A IN was less susceptible to precipitation in the presence of 2.5 μM PIR and was effectively precipitated by 5 μM PIR, the R224A and Y271A INs were comparatively more resistant to ALLINI-induced precipitation (Fig. S5).

10.1128/mbio.03560-22.6 FIG S6 Analytical size exclusion chromatography of HIV-1 IN variants. Elution chromatograms (A_280_) of full-length wild type or mutant recombinant HIV-1 IN (as indicated) from a Superdex-200 10/30 column. The expected elution volumes of the tetramer (T), dimer (D), and/or monomer (M) forms of the protein are indicated above each chromatogram. Download FIG S6, PDF file, 0.1 MB. Copyright © 2023 Singer et al. 2023 Singer et al. https://creativecommons.org/licenses/by/4.0/ This content is distributed under the terms of the Creative Commons Attribution 4.0 International license.

We also tested our panel of mutants for their effects on viral infectivity. The key CTD residues involved in the ALLINI-induced interface with the CCD are either invariant or highly conserved (Fig. 4). Unsurprisingly, the majority of mutations within the interface abrogated HIV-1 infectivity (Fig. S7). In particular, the IN mutations Y226E, W235E, K266E, I268E, and Y271A, each of which substantially compromised the ability of PIR to induce IN precipitation (Fig. S5), resulted in a loss of infectivity. These results are in concordance with earlier observations that mutations of conserved residues within HIV-1 IN CTD can cause severe replication defects (30, 48). Of the viruses we tested, only those with Q95A, W131A, and R224A mutations retained infectivity, with W131A showing partial (approximately fourfold) resistance to PIR (Fig. S7).

10.1128/mbio.03560-22.7 FIG S7 Effects of amino acid substitutions within the ALLINI-induced CTD-CCD interface on HIV-1 infectivity and PIR antiviral activity. Left panel: relative infectivity of HIV-1 variants carrying the indicated mutations within IN. The infectivity of wild type HIV-1_NL4-3_ was considered to be 100%. The bars indicate the mean values. The error bars are the standard deviations from *n* = 3 biological replicates for each condition. The open circles correspond to individual measurements. Right panel: PIR 50% effective inhibitory concentrations (EC_50_) on the wild type and HIV-1 IN mutants. Download FIG S7, PDF file, 0.02 MB. Copyright © 2023 Singer et al. 2023 Singer et al. https://creativecommons.org/licenses/by/4.0/ This content is distributed under the terms of the Creative Commons Attribution 4.0 International license.

## DISCUSSION

ALLINIs fill a small and relatively rigid pocket at the HIV-1 IN CCD dimer interface (8, 9, 11, 14–17) and act as molecular glue to paste a CTD from one IN protomer to the CCD dimer of another. The compounds bind the CCD dimer using common chemical functionalities (Fig. 1A), elements of which mimic the key amino acid residues at the tip of the LEDGF/p75 integrase-binding domain (Asp366 and Ile365) (8, 19). However, the apparent lack of similarity outside their CCD-binding function may appear inconsistent with the mode of action of these compounds. Herein, we asked how these diverse molecules bind the same face of the CTD. Our cocrystal structures with two potent ALLINIs and accompanying computational chemistry reveal that a planar aromatic scaffold is the key feature that allows these small molecules to recruit the CTD. The scaffold protrudes from the CCD pocket sufficiently to nucleate the CTD-CCD interface by engaging a triad of invariant HIV-1 IN CTD residues in a network of π-π stacking, cation-π, and hydrophobic interactions. Remarkably, while the CTD does not undergo substantial conformational changes to achieve a geometric fit to the CCD dimer (Fig. S2C), the CTD-CCD interface has not been observed within characterized functional IN complexes. Hence, this likely entirely unnatural interface can be considered to be a viral Achilles heel that is templated and leveraged by ALLINIs.

The surface of the CTD recruited by the ALLINIs shows remarkable conservation among circulating HIV-1 strains (Fig. 4). Likely due to variations within the IN CCD regions, ALLINIs specifically inhibit HIV-1 lineage viruses without any evidence for affecting closely related lentiviruses, including HIV-2 (9). Yet, it is noteworthy that the triad of the CTD residues recruited by the ALLINI aromatic scaffolds is highly conserved across diverse lentiviral IN proteins (Fig. S8A). The remarkable conservation is explained by the multiple functions played by the IN CTD in the viral life cycle and during integration, in particular. Lentiviral intasomes are thought to contain as many as 12 to 16 IN subunits (6, 39, 40, 49), and the CTDs belonging to different IN chains play diverse roles, depending on their locations within the complex. For example, the CTD residues involved in binding ALLINIs pack against the extended IN NTD-CCD linker in one type of IN chain and interact with the alpha-helical IN CCD-CTD linker region in another (Fig. S8B). Although both types of CTDs interact with viral DNA, they approach it in different ways (Fig. S8B). Therefore, mutations within this patch of the CTD surface would necessitate simultaneous adaptive changes in several positions of the IN amino acid sequence. Moreover, this compact domain has been implicated in multiple HIV-1 IN functions outside the process of integration, which may additionally limit the mutational rates at some of its key positions (50–53). Amino acid substitutions within the HIV-1 IN CTD can cause class I or class II lethal phenotypes, and the replication-defective HIV-1 K266E and W235E mutant viruses (Fig. S7) were previously characterized as class II and class I, respectively (30, 48).

10.1128/mbio.03560-22.8 FIG S8 Variable roles of IN CTDs in lentiviral integration. (A) Residues corresponding to HIV-1 IN Tyr226, Trp235, and Lys266 are highly conserved among lentiviral INs. The IN CTD regions from HIV-1, HIV-2, maedi-visna virus (MVV), equine infectious anemia virus (EIAV), feline immunodeficiency virus (FIV), and bovine immunodeficiency virus (BIV) are aligned. Red and yellow boxes highlight the amino acid residues in positions that are invariant across the alignment or are conserved through a similarity of physicochemical properties, respectively. The sequence numbering and secondary structure elements (extracted from PDB entry 6T6E) that are shown above the alignment correspond to HIV-1 IN. The key HIV-1 IN residues involved in ALLINI binding are indicated with red arrowheads (Tyr226, Trp235, and Lys266). The figure was prepared using the ESPript server (https://espript.ibcp.fr) (67). (B) The IN CTD residues involved in the ALLINI-induced interface play multiple roles within the lentiviral intasome assembly. (Top panel) HIV-1 intasome core assembly (PDB entry 6PUY [6]), with protein and viral DNA chains shown as cartoons. Catalytic IN chains are colored green and cyan. The remaining IN chains are colored according to their functions within the twofold symmetric assembly. Individual IN domains (NTD, CCD, and CTD) that belong to different IN chains and are resolved in the structure are indicated. (Lower panels) Detailed view of the CTDs belonging to IN chains that are colored orange and magenta in the top panel. DNA and selected amino acid side chains are shown as sticks, with the amino acid residues labelled. Also indicated are the alpha helical CCD-CTD and an extended NTD-CCD linker of the catalytic IN subunits. Note that the triad of IN CTD residues (Tyr226, Trp235, and Lys266) have different functions within these two types of intasomal subunits. Download FIG S8, PDF file, 0.5 MB. Copyright © 2023 Singer et al. 2023 Singer et al. https://creativecommons.org/licenses/by/4.0/ This content is distributed under the terms of the Creative Commons Attribution 4.0 International license.

The strict conservation of the HIV-1 IN CTD residues involved in interactions with ALLINIs explains why the majority of the mutations associated with resistance to this class of small molecules map in and around the binding pocket on the CCD dimer, which is considerably more variable (Fig. 4) (9, 13, 14, 16, 17, 54, 55). In most instances where resistance has occurred, it has arisen from either the exclusion of the inhibitor from its principal binding pocket at the CCD dimer interface or the loss of interaction within the pocket (54). However, at least two mutations resulting in partial resistance to ALLINIs, namely, W131C and N222K (14, 16), could not be explained by the previous cocrystal structures that lacked the CTD. Indeed, neither Trp131 nor Asn222 make direct contact with ALLINIs (Fig. 2 and 3). In our structures, the CCD residue Trp131 is involved in hydrophobic and cation-π stacking interactions with CTD residue Arg224 and makes Van der Waals contact with three additional CTD residues (Asn222, Ile268, and Asp270) (Fig. 2 and 3). The extents of these interactions would be considerably reduced upon the substitution of the bulky aromatic Trp sidechain for a Cys sulfhydryl. Although our FEP analyses suggested that the contribution of the Trp131 side chain to the CTD-CCD interface may be ALLINI-dependent (Fig. 5), we observed the partial resistance of the recombinant W131A IN to aggregation in the presence of PIR (Fig. 6; Fig. S5). Concordantly, the W131A IN virus displayed reduced sensitivity to the compound (Fig. S7). Although pinpointing the mechanism of resistance associated with N222K IN will require additional work, given the Van der Waals interaction of Asn222 with Trp131, we speculate that this amino acid substitution reduces the geometric fit between the domains and thereby compromises the ability of the drug to induce the CTD-CCD interface.

Our structures reveal atomistic details of what is likely the complete HIV-1 IN-ALLINI interface, and, as such, they will be invaluable for the further development of this promising class of antiretrovirals. Most importantly, the structures will allow for the optimization of the aromatic scaffold for improved interactions with the key HIV-1 IN CTD residues uncovered here (Tyr226, Trp235, and Lys266). By improving the ALLINI-CTD interactions, it might be possible to gain activity against HIV-1 variants that are resistant to current ALLINIs due to polymorphisms or mutations within the CCD binding pocket.

## MATERIALS AND METHODS

### Recombinant proteins.

The protein construct used for the X-ray crystallography was based on HIV-1 isolate NL4-3 and contained the IN residues 220 to 288 (CTD with the amino acid substitution W243E, including the complete C-terminal tail), which were directly followed by residues 50 to 212 (CCD, with the solubilizing mutation F185K). In this inverted design, the unstructured HIV-1 IN C-terminal tail region (residues 270 to 288), together with a portion of the NTD-CCD linker (residues 50 to 55), form a flexible linker to join the globular IN domains. The amino acid sequence of the composite linker is YGKQMAGDDCVASRQDEDMHGQVD (the underlined residues are those derived from the C-terminal tail). Isolated CTD (wild type and mutants) were used in MALLS experiments (Fig. S1A) and spanned HIV-1 IN residues 220 to 270 (corresponding to the CTD and lacking the C-terminal tail). Both proteins were produced in bacteria with N-terminal hexa-histidine (His_6_)-Sumo tags. Escherichia coli BL21-CodonPlus (DE3) cells (Agilent) that were transformed with the desired recombinant expression plasmid were grown in Luria broth in shaker flasks at 30°C. Protein expression was induced via the addition of 0.01% (wt/vol) isopropyl β-D-1-thiogalactopyranoside (IPTG) to log phase cultures for 4 h. To isolate the recombinant proteins, the cells were lysed via sonication in core buffer (0.5 M NaCl and 20 mM Tris-HCl, pH 7.5) supplemented with 1 mM phenylmethylsulfonyl fluoride and cOmplete EDTA-free protease inhibitor cocktail (Roche). The supernatant, precleared by centrifugation, was incubated with Ni-NTA agarose (Qiagen) at 4°C in the presence of 15 mM imidazole for 1 h. The resin was extensively washed with core buffer supplemented with 15 mM imidazole, and the recombinant protein was eluted with 200 mM imidazole in core buffer. The eluted protein, supplemented with 2 mM dithiothreitol (DTT), was incubated with Ulp1 sumo protease (using 0.01 mg of protease per mg of recombinant protein) at 4°C for 16 h to release the His_6_-Sumo tag. The next day, the cleaved protein was diluted to adjust the NaCl concentration to 150 mM, injected into a 5-mL HiTrap SP HP column (Cytiva), and eluted with a linear 0.15 to 1.0 M NaCl gradient in 20 mM Tris-HCl, pH 7.5. The peak fractions were separated via size exclusion chromatography (SEC) through a HiLoad 16/600 Superdex 200 pg column (Cytiva) that was equilibrated in core buffer. For the SPR experiments, the removal of the His_6_-Sumo tag was omitted.

Full-length wild type and mutant HIV-1 IN proteins were expressed in E. coli BL21(DE3) cells and purified via sequential chromatography on nickel and heparin affinity columns as described (56, 57). Isolated CCD constructs spanning HIV-1 IN residues 50 to 212 with the solubilizing mutation F185H and an N-terminal His_6_ tag were produced in E. coli as described (57). The protein was purified via chromatography on a nickel affinity column, and this was followed by SEC using a HiLoad 16/600 Superdex column (Cytiva), with the elution buffer containing 0.5 M NaCl, 20 mM HEPES (pH 7.5), 10% glycerol, 0.5 mM EDTA, and 2 mM DTT.

### SEC-MALLS.

Size exclusion chromatography coupled to multiangle laser light scattering (SEC-MALLS) was used to determine the molar mass distribution of the wild type and mutant HIV-1 IN CTDs. Samples (100 μL) were applied to a Superdex-75 Increase 10/300 column (Cytiva) that was equilibrated in 0.5 M NaCl, 25 mM Tris-HCl (pH 7.5), 3 mM NaN_3_ and 0.5 mM TCEP and was mounted on a JASCO-4000 semimicro HPLC system. Chromatography was performed at 25°C, at a flow rate of 1 mL/min. The scattered light intensities and protein concentrations of the eluted peaks were recorded using a DAWN-HELEOS II laser photometer and an OPTILAB-TrEX differential refractometer (Wyatt Technology), respectively. The weight-averaged molar mass of the material contained in the chromatographic peaks was determined using the combined data from both detectors and *dn/dc *= 0.186 mL/g in the ASTRA software version 7.3.2 (Wyatt Technology).

### ALLINIs.

BI-D was obtained from MedChemExpress (product number HY-18601). PIR was synthesized as previously described (58) and verified via mass spectrometry (*M*_r_ = 495.1 Da).

### Surface plasmon resonance.

SPR studies were performed using a four-channel Reichert4 SPR instrument (AMETEK, Reichert Technologies). His_6_-tagged CCD (F185H) and (His_6_)-Sumo-CTD-CCD (F185K/W243E) were captured onto an NTA chip to about 4,500 response units, using HBS-*P*+ buffer containing 10 mM HEPES, 150 mM NaCl, and 0.05% (vol/vol) surfactant P20 (Cytiva) supplemented with 5% dimethyl sulfoxide (DMSO). A method development capture assay was executed to establish the best association and dissociation times for both the ligand and the analyte. After the determination of the parameters, experiments were carried out in triplicate for each ligand. The data were analyzed using the Reichert4SPR and Tracedrawer software packages to calculate the *k_on_*, *k_off_*, and *K_D_* values via a 1:1 binding model.

### Analytical SEC.

The self-association of recombinant wild type and mutant INs was analyzed on a Superdex 200 10/300 GL column (GE Healthcare). The column was equilibrated in a running buffer that contained 20 mM HEPES (pH 7.5), 1 M NaCl, 10% (vol/vol) glycerol, 7.5 mM 3-([3-cholamidopropyl] dimethylammonio)-1-propanesulfonate, and 5 mM β-mercaptoethanol at a flow rate of 0.45 mL/min. The proteins were diluted to 20 μM with the running buffer and incubated for 1 h at 4°C. This was followed by centrifugation at 10,000 × *g* for 20 min. The retention volumes for the IN tetramers, dimers, and monomers were approximately 12.3 mL, 14.0 mL, and 15.0 mL, respectively.

### Protein crystallization.

Stock solutions of BI-D and PIR were prepared for the cocrystallization experiments in DMSO at concentrations of 50 mM and 60 mM, respectively. To prepare the IN-ALLINI complexes, W243E/F185K CTD-CCD (0.6 mg/mL in core buffer) was supplemented with 25 μM ALLINI (diluted directly from the stocks in DMSO) in the absence (BI-D) or presence (PIR) of 5% (vol/vol) glycerol. Following incubation for 10 min on ice, the IN-ALLINI complexes were concentrated to 5 mg/mL using a 10-kDa cutoff VivaSpin device (Sigma-Aldrich). Crystals were grown at 18°C in hanging drops that were formed by combining 1 μL of protein with 1 μL of a reservoir solution, which contained either 10% polyethylene glycol (PEG) 4,000, 17.5% glycerol, 30 mM MgCl_2_, 30 mM CaCl_2_, and 0.1 M Tris-Bicine (pH 8.5) (BI-D cocrystals) or 10% (wt/vol) PEG 8,000, 20% ethylene glycol, 30 mM MgCl_2_, 30 mM CaCl_2_, and 0.1 M imidazole-MES (pH 6.5) (PIR cocrystals). The crystals, which were cryoprotected in mother liquor supplemented with 30% glycerol, were flash-frozen via plunging into liquid nitrogen.

### X-ray data collection and structure refinement.

The diffraction data were collected at beamline I04 of the Diamond Light Source (Oxford, UK) at 100 K, using a wavelength of 0.9795 Å, a transmission of 100%, and a 43 × 30 μm beam with either 0.1-s exposure and 0.2° rotation per image (BI-D cocrystals) or 0.3-s exposure and 0.5° rotation per image (PIR cocrystals). The single-crystal diffraction data were integrated, scaled, and merged with DIALS (59) within the Xia2 automatic data processing pipeline (60). The structures of the HIV-1 IN CTD (PDB entry 6T6E [47]) and CCD (PDB entries 4O55 [11] and 2B4J [19]) were used as the search models for molecular replacement in PHASER (61) within the Phenix software suite (62). The resulting models were adjusted with multiple rounds of interactive fitting in Coot (63) and refinement using phenix.refine (version 1.20.1-4487-000) (62, 64). Protein chains were initially extended where possible, and then water, PEG, glycerol, and/or ethylene glycol molecules were added, as is visualized in the electron density maps. ALLINI molecules were then fitted into prominent positive difference densities. The ligand geometry definition files were generated using eLBOW (65). The final refinements included 5 translation/libration/screw (TLS) anisotropic B factor groups per protein chain, guided by the TLSMD server (66). The refined models fit well into the electron density and had good geometry, as assessed by the MolProbity server (http://molprobity.biochem.duke.edu) (66) (Table S1; Fig. S1C). The crystal structure illustrations were prepared using PyMol software (https://pymol.org), and the IN amino acid sequence alignment (Fig. S8A) was formatted using ESPript (https://espript.ibcp.fr) (67).

### Parameterization of ALLINI molecules.

The calculation of the atomic interaction potentials for molecular dynamics requires empirical parameters for every bond, angle, dihedral, and Van der Waals interaction. We obtained the initial parameters for BI-D from the CHARMM General Force Field (CGenFF) (68). Further, initial parameters with an assigned penalty higher than 10 (Fig. S9A) were optimized using the VMD Force Field Toolkit (ffTK) (69) and Gaussian-16 (70). For this process, to reduce the computational cost of the calculations, a divide and conquer approach was applied. First the BI-D molecule was fragmented into three moieties (Fig. S9C and D). Then, the fragments were chosen to span the dihedral angles to be parameterized and to facilitate the reassembly of the full molecule. The geometry of each fragment was first optimized using Gaussian at a MP2/6-31* level of theory under water solvent to ensure that no hydrogen bonds were generated between parts of the same moiety. Then, the partial charge of each water-accessible atom was optimized to reproduce its role as a hydrogen bond acceptor or donor, following the CHARMM conventions. A water molecule was placed 2 Å apart from the atom of interest with either a hydrogen atom (for the hydrogen bond acceptors) or the oxygen atom (for the hydrogen bond donors) of water oriented toward the atom. The distance and orientation of the water-molecule complex was optimized quantum mechanically in Gaussian at the b3lyp/6-31g(d,p) level of theory. These were then transferred to ffTK to fit the atomic partial charges. The bond and angle parameters were optimized in ffTK by comparing the molecular mechanics (MM) potential energy distribution (PES) with the quantum mechanics PES that was derived from the Hessian matrix calculation for each fragment calculated in Gaussian at the MP2/6-31G* theory level. Finally, the dihedral parameters were optimized by calculating the torsion angle scans (MP2/6-31G*) and fitting the MM PES to reproduce the maxima and minima. The resulting MM PES and QM PES from our parameterization (Fig. S9B) yielded a root mean square difference between the energy profiles of 1.44 kcal/mol. Once all of the high penalty parameters were optimized from the fragments, the optimized partial charges that were assigned to the original molecule by moving the charges of overlapping atoms to their closest nonoverlapping neighbors were used to conserve the total charge of the molecule. The force field parameters for PIR were obtained from CGenFF and used as is, since we observed that they reproduce the stability of this molecule in the binding pocket.

10.1128/mbio.03560-22.9 FIG S9 Parameterization scheme and molecular fragmentation of BI-D. (A) Dihedral angle parameters with CGenFF penalties above 10. (B) Parameterized molecular mechanics potential (solid blue line) fitted to the quantum chemistry calculated potential (dotted black line). The rms difference between both potentials was 1.44 kcal/mol. (C) 3D model of BI-D. (D) To perform dihedral scans in the parameterization scheme, the molecule was fragmented into three moieties, and these are shown as stick diagrams (left) and 3D models (right). Download FIG S9, PDF file, 0.2 MB. Copyright © 2023 Singer et al. 2023 Singer et al. https://creativecommons.org/licenses/by/4.0/ This content is distributed under the terms of the Creative Commons Attribution 4.0 International license.

### Molecular dynamics setup.

We initiated all of the molecular dynamics (MD) and free energy perturbation (FEP) simulations of the HIV-1 IN structures with bound BI-D or PIR from the crystal structures that are presented in the manuscript. Additionally, we conducted MD simulations of an *apo* conformation of the HIV-1 IN, which was obtained by removing the BI-D molecules from the binding pockets. Before conducting the simulations, we modeled and added the following loop residues, which were missing from the crystal structure of the HIV-1 IN in the presence of BI-D: residues 140 to 148 and 189 to 192 (chain A), residues 141 to 148 and 189 to 192 (chain B), residues 230 to 231 (chain C), and residues 230 to 233 (chain D). The following missing loop residues were modeled and added to the crystal structure of the HIV-1 IN in the presence of PIR: residues 145 to 148 (chain A) and residues 141 to 147 (chain B). The modeling of the missing residues was performed using the Modeller software (Fig. S3A) (71). We retained two Mg^2+^ ions from the crystal structures. Hydrogen atoms were added to the HIV-1 IN, BI-D, and PIR inhibitors while retaining the (S) form chirality of the BI-D and PIR inhibitors, as the (S) form was determined to be more predominant and bioactive than was the (R) form for these compounds (17, 72). Further, we solvated each system in a periodic box of TIP3P water molecules and ionized them with Na^+^ and Cl^−^ ions to a salt concentration of 150 mM (Fig. S3B and D) using VMD software (73). The final simulation domains contained approximately between 132,000 and 142,000 atoms, with an overall system size of approximately 116 Å × 109 Å × 120 Å.

Each simulation domain was equilibrated in the presence and absence of inhibitors using NAMD2.14 simulation software (74), according to the following procedure. In the first step, we applied restraints to the protein and inhibitors while the solvent molecules were energy minimized using the conjugate gradient scheme until the gradient converged to values below 10 kcal mol^−1 ^Å^−1^. In the next step, the restraints on the protein and inhibitors were released, and the system was energy minimized using the conjugate gradient scheme until the gradient converged to values below 10 kcal mol^−1 ^Å^−1^. Each minimization was followed by the tempering of the simulation domain from 50 K to 310 K in increments of 5 K over 0.5 ns. During the second tempering step, we applied positional restraints to the heavy backbone atoms of the HIV-1 IN and to the heavy atoms of the inhibitors with a force constant of 10 kcal mol^−1 ^Å^−1^. Subsequently, we performed the equilibration simulations of each system while the positional restraints on the heavy backbone atoms of the HIV-1 IN and on the heavy atoms of the inhibitors were gradually released at a rate of 1 kcal mol^−1 ^Å^−1^ per 0.5 ns from 10 kcal mol^−1 ^Å^−1^ to 0 kcal mol^−1 ^Å^−1^ over 5 ns. During the equilibration simulations, we maintained the temperature at 310 K with a coupling constant of 1 ps^−1^, using a Langevin thermostat. The pressure was maintained at 1 atm, with the period and decay parameters set to 100 ps and 50 ps, using a Nose-Hoover barostat.

After performing the equilibration simulations, we conducted production MD and FEP simulations in the *NVT* ensemble with the temperature set to 310 K and the coupling constant set to 1 ps^−1^. These were maintained using a Langevin thermostat. We used periodic boundary conditions in all of the MD and FEP simulations with a 2 fs timestep. The electrostatic interactions were calculated using the particle mesh Ewald method with the cutoff for short-range electrostatics interactions being set to 12 Å. The coordination number between two Mg^2+^ ions and protein atoms which were located within a cutoff of 5 Å from the Mg^2+^ ions was constrained in all of the simulations by using the *coordNum* function in the Colvar module (75) of NAMD. We conducted all of the simulations using the CHARMM36m force field for proteins (76), the Roux force field parameters for ions (77), and the TIP3P water model (78). We derived the force field parameters for the BI-D inhibitor by using quantum mechanical calculations in conjunction with the CGenFF2.5 software package (79). The force field parameters for PIR were determined solely using the CGenFF2.5 software package with the CGenFF force field version 4.5 (68, 80). For each system (without or with ALLINI), we generated a 1 μs MD simulation, which resulted in an overall 3 μs data set and saved frames every 10 ps. An all-to-all rms deviation matrix analysis was performed as described (81).

### Free energy perturbation (FEP) protocol.

We determined the relative free energy differences (ΔΔG) associated with the mutations of selected amino acid residues that comprise the binding pockets of BI-D and PIR inhibitors via the *in silico* transformation of wild type residues into alanine using the FEP method (82) in conjunction with a thermodynamic cycle (Fig. S3E). FEP is an old but commonly used technique (83) to compute free energy differences, which has previously been successfully applied to determine the energetics of RNA/protein interactions (84, 85), protein/protein interactions (86, 87), ligand/protein interactions (88, 89), and ligand/DNA interactions (90), as well as to address other scientific problems (91). In this work, we conducted alchemical transformations that correspond to the horizontal arms in the thermodynamic cycle (Fig. S3E), both in the unbound HIV-1 IN (free state) and in the bound HIV-1 IN with BI-D or PIR molecules (complex state). The vertical arms in the thermodynamic cycle represent the binding of BI-D or PIR to IN (Fig. S3E). We calculated the relative free energy differences according to $\Delta \Delta G=\Delta G^{\mathrm{comp}}-\Delta G^{\mathrm{free}}=\Delta G_{\mathrm{wt}}^{\mathrm{bind}}-\Delta G_{\mathrm{mut}}^{\mathrm{bind}}$, in which $\Delta G^{\mathrm{comp}}$ and $\Delta G^{\mathrm{free}}$ correspond to free energy changes along the horizontal arms of the thermodynamic cycle, and $\Delta G_{\mathrm{wt}}^{\mathrm{bind}}$ and $\Delta G_{\mathrm{mut}}^{\mathrm{bind}}$ correspond to the free energy changes along the vertical arms of the thermodynamic cycle and characterize the binding of the ALLINIs to the wild type HIV-1 IN and mutant HIV-1 IN (Fig. S3E).

We applied the dual-topology paradigm (92), in which a hybrid energy function (U) is utilized to represent a mixture of the initial and final states of one of the horizontal arms of the thermodynamic cycle. The initial and final states were connected by a coupling parameter (λ) that corresponds to a series of individual simulations of the intermediate states (93). The coupling parameter spans values between λ = 0 and λ = 1, which correspond to the physical initial and final states, respectively, and the intermediate values correspond to mixed unphysical states. Each window of the alchemical transformation was prefaced by 0.2 ns of equilibration of the simulation domain, and this was followed by 0.8 ns of data generation for the free energy calculations.

We used the bidirectional approach (94) in each alchemical transformation by propagating the simulations in both the forward and backward directions to improve the accuracy of the free energy calculations. We determined the free energy changes ($\Delta G^{\mathrm{free}}$ and $\Delta G^{\mathrm{comp}}$) with their associated statistical errors over the forward and backward simulations with 20 equally spaced λ windows by using the Bennet acceptance ratio estimator (95) which is implemented in the ParseFEP plugin (96) in VMD1.9.3. Each transformation was repeated in quadruplicate, and the resulting free energy changes were averaged to yield the final free energy difference. All of the free energy differences, along with the final free energy difference for each alchemical transformation, are reported in Table S1. We conducted all of the FEP simulations using NAMD3.0 simulation software (74).

We computed the heavy atom (C, N, O, and S) rms deviation for the CCD and CTD with respect to the initial frame of the corresponding simulation in each system to establish the effects of the ALLINIs on the protein domain structure. The calculated rms deviation values indicate changes in the CCD or CTD with respect to a reference state. We also computed the per-residue rms fluctuations based on the alpha carbons in the protein backbone to further investigate the flexibility of each amino acid and each protein domain.

### HIV-1 IN aggregation assays.

For DLS, wild type and mutant IN proteins were diluted from 200 nM stocks to 12.5 nM in buffer containing 1 M NaCl, 2 mM MgCl_2_, 2 mM DTT, and 50 mM HEPES-NaOH (pH 7.5) that were prepared with Milli-Q grade water and filtered twice with a 0.2 μm filter. PIR (62.5 nM) was added, and particle size distributions for the wild type and for each protein were recorded over a course of 10 min at 1 min intervals using a Zetasizer Nano ZS90 instrument (Malvern Panalytical). Any signal below a 2 nm threshold was considered to be background.

To observe ALLINI-induced precipitation, 4 μM wild type or mutant IN was incubated with increasing concentrations of PIR in 20 μL of assay buffer that contained 1 M NaCl, 5 mM β-mercaptoethanol, and 20 mM HEPES-NaOH (pH 7.5) at 4°C overnight. Following centrifugation at 10,000 × *g* for 10 min, the supernatant was mixed with 100 μL of 1.25× Novex NuPAGE LDS sample buffer. The remaining pellet was washed 3 times with 40 μL of assay buffer before resuspension in 120 μL of Novex NuPAGE LDS sample buffer to match the overall volume of the corresponding supernatant fraction. The supernatant and pellet fractions were analyzed via SDS-PAGE, and the IN was visualized via staining with Coomassie blue.

### Virus infectivity assay.

No cell lines from the list of known misidentified cell lines that is maintained by the International Cell Line Authentication Committee were used in this study. HEK293T (American Type Culture Collection) and HeLa TZM-bl (NIH AIDS Reference and Reagent Program) cells were cultured in Dulbecco’s modified Eagle’s medium (DMEM, Gibco) that was supplemented with 10% fetal bovine serum (Sigma-Aldrich), 100 U/mL penicillin, and 100 U/mL streptomycin (Thermo Fisher Scientific). The cell lines were maintained at 37°C in a humidified 5% CO_2_ atmosphere, and they were tested monthly for *Mycoplasma* contamination. For the virus infectivity assays, HEK293T cells were seeded at concentrations of 2 to 4 × 10^5^ cells/mL in 6-well plates 1 d prior to transfection with 2 μg of replication-competent pNL4-3 (wild type or IN mutants), using the HilyMax transfection reagent (Dojindo Molecular Technologies) in a 1:3 ratio, following the manufacturer’s protocol. The medium was replaced with fresh medium at 12 to 16 h posttransfection and incubated at 37°C. 48 h posttransfection, viruses containing clarified supernatant were filtered through a 0.45 μm filter, and the level of p24 (CA) was quantified via Western blotting. The level of CA was similar in all viruses, so 50 μL of filtered viral supernatant from the HEK293T cells were used to infect the TZM-bl cells (seeded at 50,000 cells/well) and incubated at 37°C for 3 to 4 h. Then, the medium was removed and replaced with fresh medium. The cells were collected at 48 h postinfection, and the virus infectivity was measured via a luciferase assay (Promega).

To test the STP-0404 antiviral activity, full replication cycle experiments (wild type or IN mutants) were performed as described previously (11, 97). Briefly, viruses were prepared from HEK293T cells in the presence of various concentrations of STP-0404 or with DMSO alone as a control. Target HeLa TZM-bl cells were similarly preincubated with STP-0404 or with DMSO. The cells were then infected with viruses for 3 to 4 h at 37°C, the medium was replaced, and fresh inhibitors were added. 48 h postinfection, the cells were harvested, and the infectivity was measured via a luciferase assay. The STP-0404 EC_50_ values were calculated using Origin software (OriginLab). All of the virus infections were performed in the presence of 8 μg/mL Polybrene, and the values are expressed as the mean ± the standard deviation (SD).

### Data availability.

The data that support this study are available from the corresponding authors upon reasonable request. The refined models and the associated X-ray diffraction data were deposited into the Protein Data Bank under accession codes 8A1P (BI-D cocrystal structure) and 8A1Q (PIR).
