# Supplementary material for: The Drug-Induced Interface That Drives HIV-1 Integrase Hypermultimerization and Loss of Function
Source: mBio. 2023 Feb 6;14(1):e03560-22. doi: 10.1128/mbio.03560-22 (PMC9973045; doi:10.1128/mbio.03560-22)

A

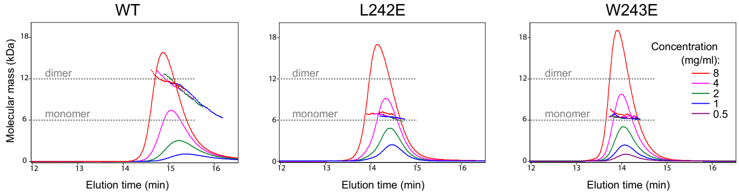

B

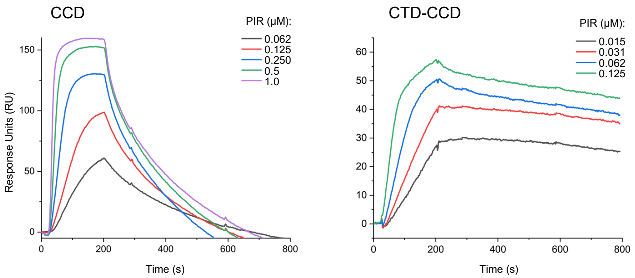

	K_{on} (M^{-1}. s^{-1})	K_{off} (s^{-1})	K_D (nM)
**CCD**	**4.86 ± 0.86 x 10^4**	**6.76 ± 0.76 x 10^{-3}**	**139 ± 8**
**CTD-CCD**	**1.52 ± 0.44 x 10^5**	**5.9 ± 2.54 x 10^{-4}**	**4.74 ± 3.04**
**Fold change**	**~3**	**~ 10**	**~29**

C

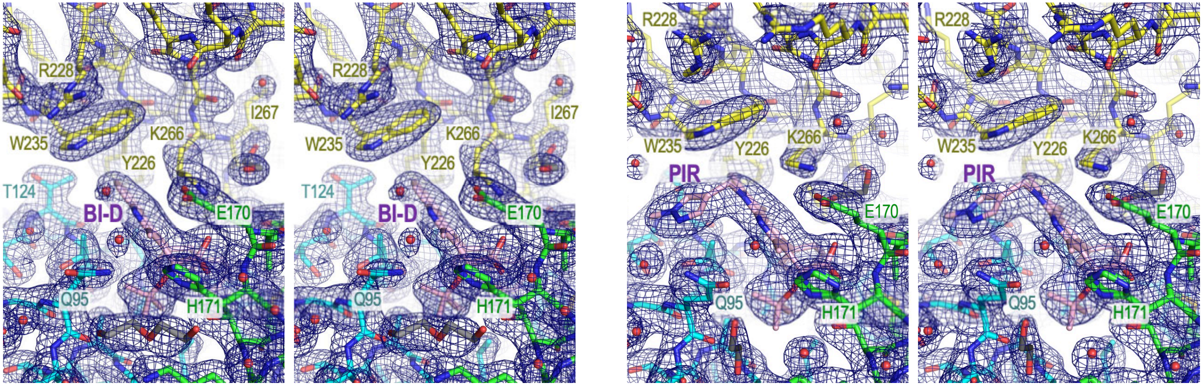

Supplement: FIG S1 [file mbio.03560-22-s0001.pdf]
