# Supplementary figures and images for: The Drug-Induced Interface That Drives HIV-1 Integrase Hypermultimerization and Loss of Function
Source: mBio. 2023 Feb 6;14(1):e03560-22. doi: 10.1128/mbio.03560-22 (PMC9973045; doi:10.1128/mbio.03560-22)

A

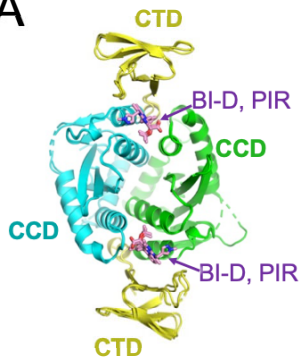

B

PDB ID 5HOT:

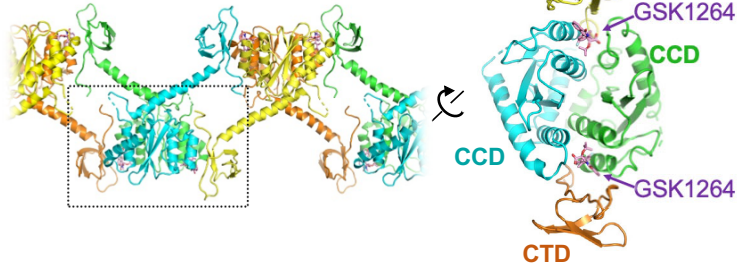

C

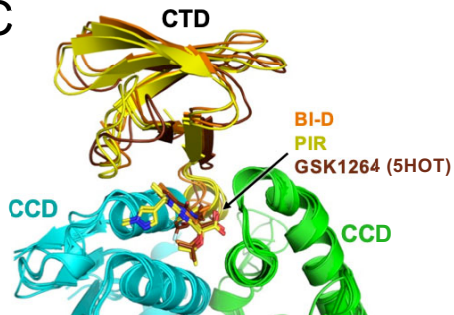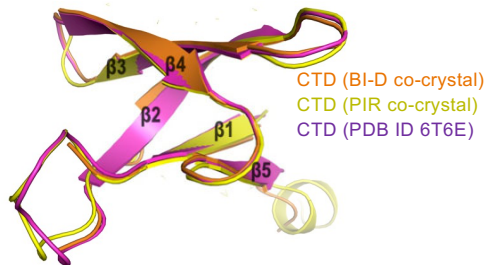

Supplement: FIG S2 [file mbio.03560-22-s0002.pdf]

A

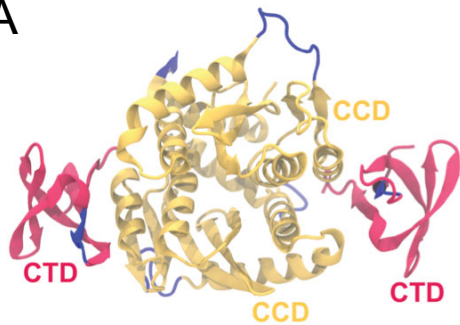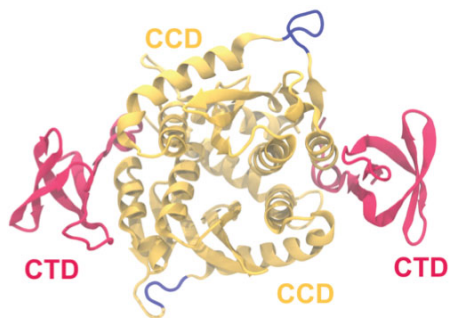

B

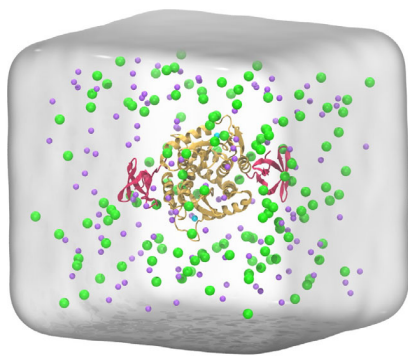

C

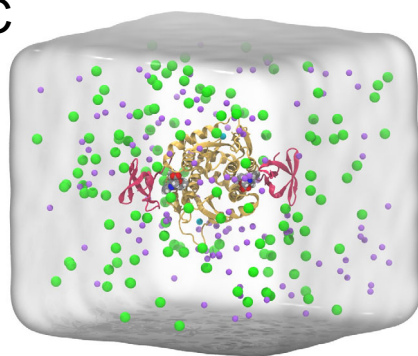

D

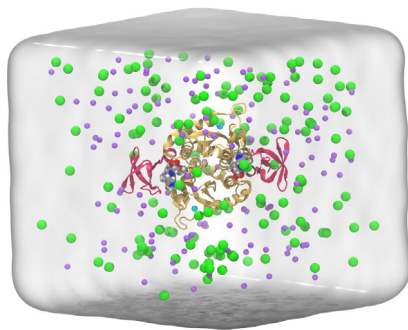

E

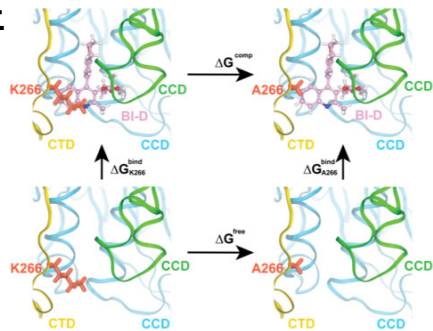

Supplement: FIG S3 [file mbio.03560-22-s0003.pdf]

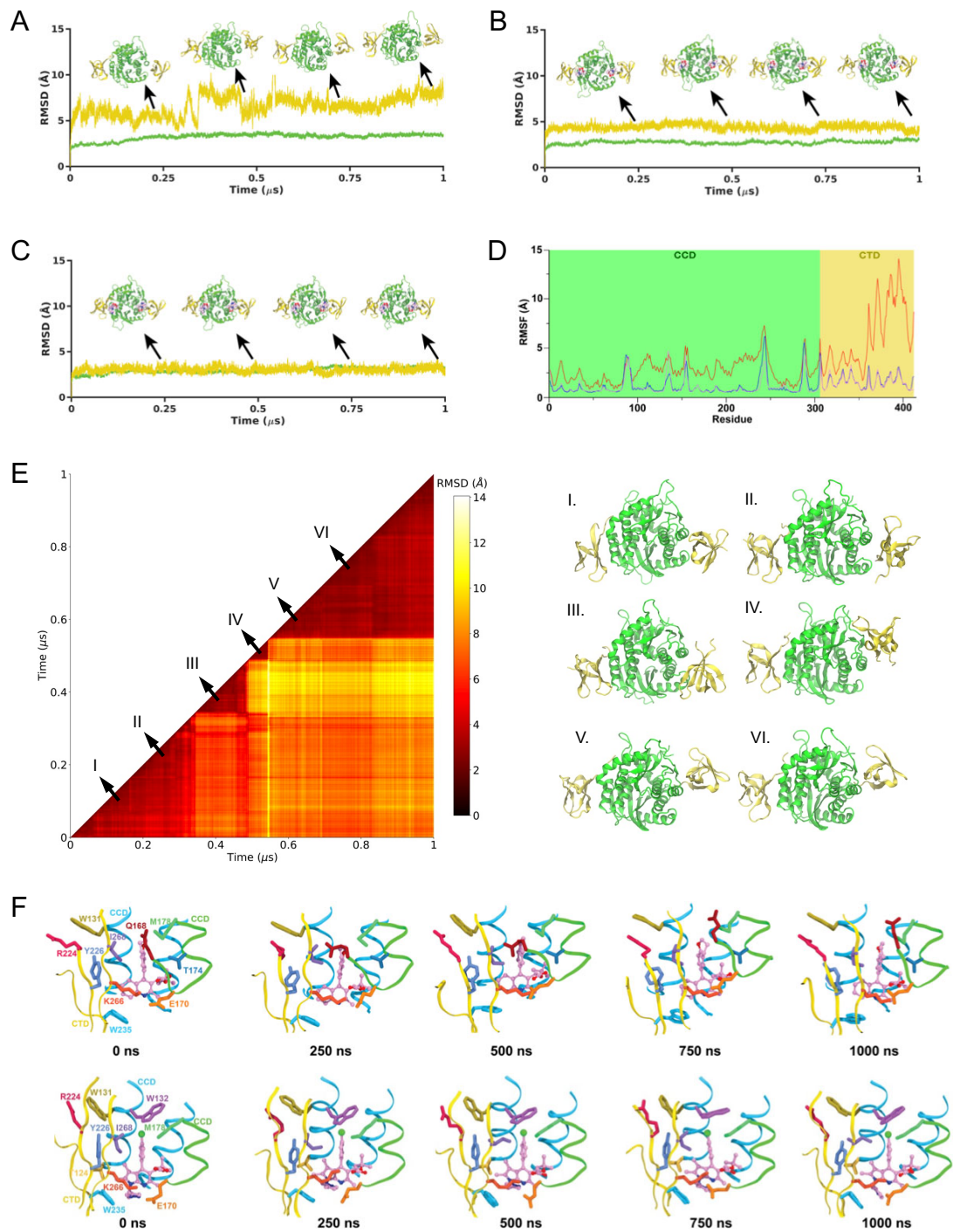

Supplement: FIG S4 [file mbio.03560-22-s0004.pdf]

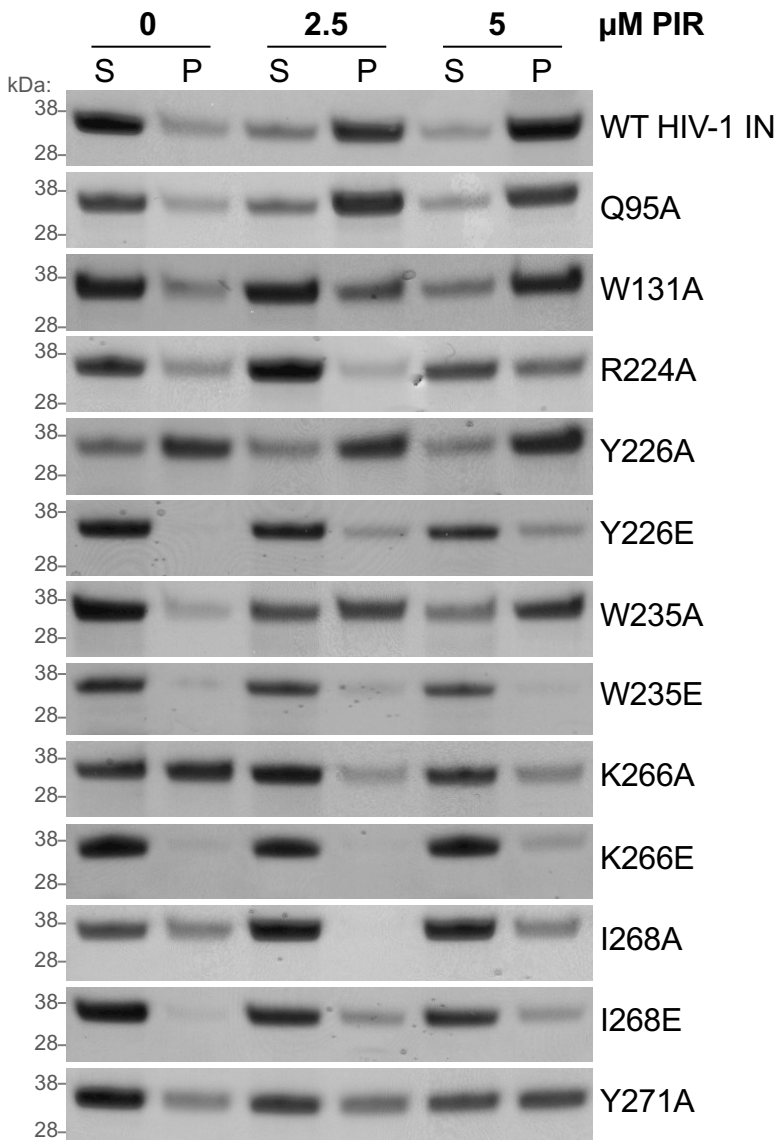

Supplement: FIG S5 [file mbio.03560-22-s0005.pdf]

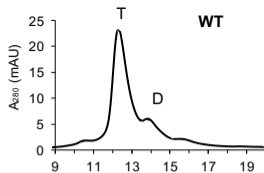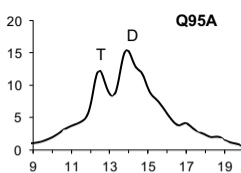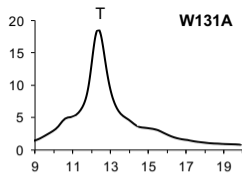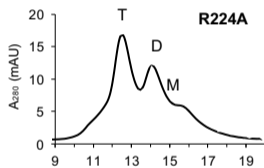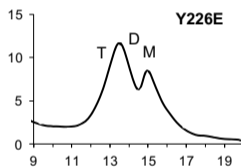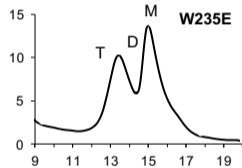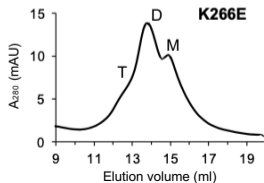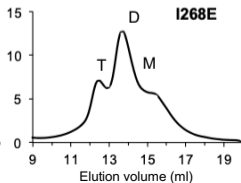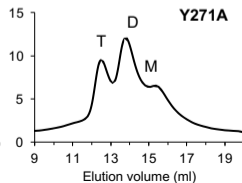

Supplement: FIG S6 [file mbio.03560-22-s0006.pdf]

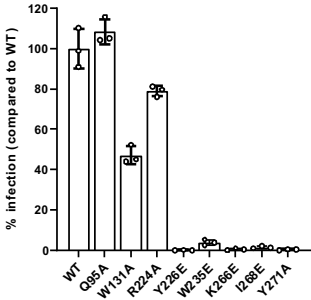

| HIV-1 <sub>NL4-3</sub> | PIR EC <sub>50</sub> (nM) |
|------------------------|---------------------------|
| IN(WT)                 | 12.1 ± 0.4                |
| IN(Q95A)               | 10.1 ± 1.5                |
| IN(W131A)              | 45.1 ± 4.4                |
| IN(R224A)              | 7.2 ± 0.5                 |

Supplement: FIG S7 [file mbio.03560-22-s0007.pdf]

**A****Charges****Dihedrals**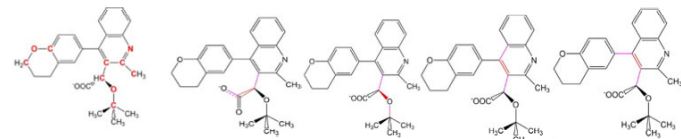**Bonds****Angles****None**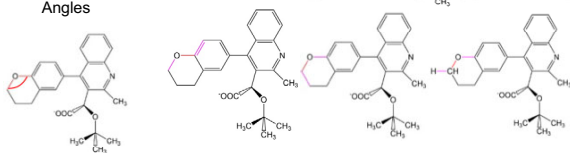**B**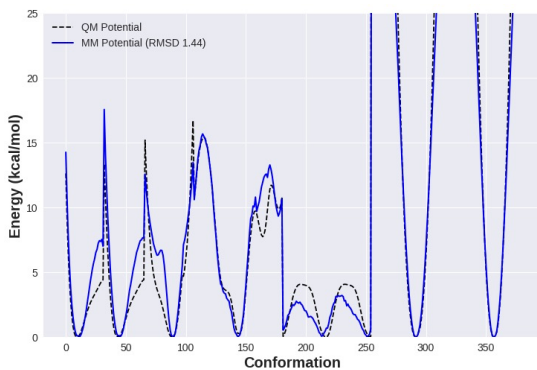**C**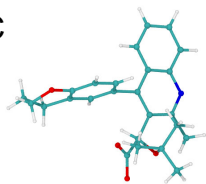**D**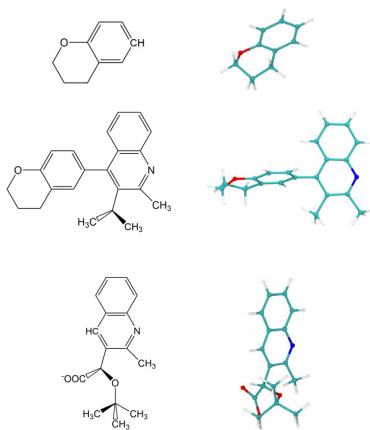

Supplement: FIG S9 [file mbio.03560-22-s0009.pdf]
