# Supplementary material for: The Drug-Induced Interface That Drives HIV-1 Integrase Hypermultimerization and Loss of Function
Source: mBio. 2023 Feb 6;14(1):e03560-22. doi: 10.1128/mbio.03560-22 (PMC9973045; doi:10.1128/mbio.03560-22)
Supplement: FIG S8 [file mbio.03560-22-s0008.pdf]

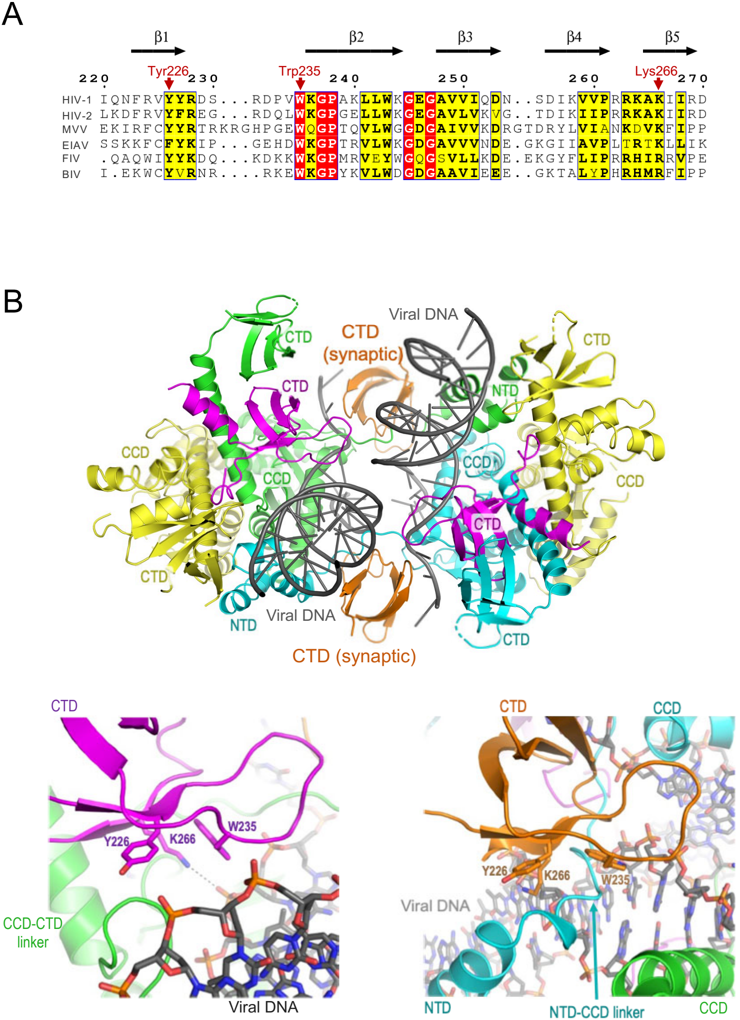
A
β1
β2
β3
β4
β5
220
Tyr226
230
Trp235
240
250
260
Lys266
270
HIV-1 IQNFRVYYRDS...RDPVWKGPAKLLWKGEGAVVIQDN..SDIKVVPRRKAKIIRD
HIV-2 LKDFRVYFREG...RDQLWKGPGELLWKGEGAVLVKVG..TDIKIIPRRKAKIIRD
MVV EKIRFCYYRTRKRGHPGEWQGPTQVLWGGDGAIVVKDRGTDRYLVIANKDVKFIPP
EIAV SSKKFCFYKIP...GEHDWKGPTRVLWKGDGAVVVNDE.GKGIIAVPLTRTKLLIK
FIV .QAQWIYYKDQ...KDKKWKGPMRVEYWGQGSVLLKDE.EKGYFLIPRRHIRRVPE
BIV I.EKWCYVRNR....RKEWKGPYKVLWDGDGAAVIEEE..GKTALYPHRHMRFIPP
B
Viral DNA
CTD (synaptic)
CTD
CTD
NTD
CTD
CCD
CCD
CCD
CCD
CTD
CTD
NTD
Viral DNA
CTD
CTD (synaptic)
CTD
W235
Y226
K266
CCD-CTD linker
Viral DNA
CTD
CCD
Y226
K266
W235
Viral DNA
NTD
NTD-CCD linker
CCD
